# Non-invasive mapping of systemic neutrophil dynamics upon cardiovascular injury

**DOI:** 10.1038/s44161-022-00210-w

**Published:** 2023-02-06

**Authors:** Pascal Bouvain, Zhaoping Ding, Shiwa Kadir, Patricia Kleimann, Nils Kluge, Zeynep-Büsra Tiren, Bodo Steckel, Vera Flocke, Ria Zalfen, Patrick Petzsch, Thorsten Wachtmeister, Gordon John, Nirojah Subramaniam, Wolfgang Krämer, Tobias Strasdeit, Mehrnaz Mehrabipour, Jens M. Moll, Rolf Schubert, Mohammad Reza Ahmadian, Florian Bönner, Udo Boeken, Ralf Westenfeld, Daniel Robert Engel, Malte Kelm, Jürgen Schrader, Karl Köhrer, Maria Grandoch, Sebastian Temme, Ulrich Flögel

**Affiliations:** 1 Experimental Cardiovascular Imaging, Institute for Molecular Cardiology, Heinrich Heine University, Düsseldorf, Germany; 2 Biological and Medical Research Center (BMFZ), Medical Faculty, Heinrich Heine University, Düsseldorf, Germany; 3 Dental Office/Oral Surgery, Dr. G. John, Plauen, Germany; 4 Institute for Experimental Immunology and Imaging, Department of Immunodynamics, University Hospital Essen, University Duisburg-Essen, Essen, Germany; 5 Department of Pharmaceutical Technology and Biopharmacy, Albert Ludwig University, Freiburg im Breisgau, Germany; 6 Institute of Neuro- and Sensory Physiology, Heinrich Heine University, Düsseldorf, Germany; 7 Institute of Biochemistry and Molecular Biology II, Medical Faculty and University Hospital Düsseldorf, Heinrich Heine University, Düsseldorf, Germany; 8 Department of Cardiology, Pneumology and Angiology, University Hospital Düsseldorf, Düsseldorf, Germany; 9 Clinic for Cardiac Surgery, University Hospital Düsseldorf, Düsseldorf, Germany; 10 Cardiovascular Research Institute Düsseldorf (CARID), Heinrich Heine University, Düsseldorf, Germany; 11 Institute for Translational Pharmacology, Heinrich Heine University, Düsseldorf, Germany; 12 Department of Anesthesiology, University Hospital Düsseldorf, Düsseldorf, Germany

**Keywords:** Biomedical engineering, Preclinical research, Neutrophils, Molecular imaging

## Abstract

Neutrophils play a complex role during onset of tissue injury and subsequent resolution and healing. To assess neutrophil dynamics upon cardiovascular injury, here we develop a non-invasive, background-free approach for specific mapping of neutrophil dynamics by whole-body magnetic resonance imaging using targeted multimodal fluorine-loaded nanotracers engineered with binding peptides specifically directed against murine or human neutrophils. Intravenous tracer application before injury allowed non-invasive three-dimensional visualization of neutrophils within their different hematopoietic niches over the entire body and subsequent monitoring of their egress into affected tissues. Stimulated murine and human neutrophils exhibited enhanced labeling due to upregulation of their target receptors, which could be exploited as an in vivo readout for their activation state in both sterile and nonsterile cardiovascular inflammation. This non-invasive approach will allow us to identify hidden origins of bacterial or sterile inflammation in patients and also to unravel cardiovascular disease states on the verge of severe aggravation due to enhanced neutrophil infiltration or activation.

## Abstract (web summary)

Bouvain et al. develop fluorine-loaded nanotracers engineered with binding peptides targeting the neutrophil-specific receptor CD177 for specific and non-invasive mapping of murine and human neutrophil dynamics by whole-body MRI.

## Main

Neutrophils are an important part of the innate immune system^1^ and play a crucial role in host defense against infections. They contribute not only to the development and progression of sterile inflammation in atherosclerosis but also to the healing process after ischemic insults such as stroke and myocardial infarction (MI)^2^. During inflammatory challenges, they are rapidly released from the bone marrow into the blood, which can lead to a tenfold increase in circulating neutrophils. Subsequently, neutrophils are recruited into inflamed areas where they internalize pathogens and cell debris, release reactive oxygen species (ROS) or generate nuclear extracellular traps^3^. Tracking of neutrophils by optical techniques has provided insight into new functions of neutrophils such as reverse transendothelial migration and tissue-specific recruitment mechanisms^4–6^. These methods are characterized by high sensitivity and spatial resolution, but systemic and non-invasive in vivo mapping with sufficient tissue penetration to monitor neutrophil trafficking from their origin in the bone marrow to the injured target organ was not feasible thus far.

Among the molecular imaging techniques capable of whole-body scanning, lately fluorine (^19^F) MRI has emerged as a promising tool^7^. Fluorine-19 offers high sensitivity and is nearly absent from biological tissue. Thus, accumulation of ^19^F gives rise to ‘hot spots’ without any natural background that can be merged with anatomical ^1^H datasets to assess their location. To generate ^19^F-based MRI probes, we made use of emulsified, biochemically inert perfluorocarbons (PFCs), which are characterized by a very high payload of ^19^F. After intravenous injection, ‘neat’ PFCs are readily taken up by phagocytic immune cells, which has already been reported as a side effect during their clinical exploration as an artificial blood substitute^8^. Although neutrophils can also be labeled to a certain degree by conventional PFCs as bystander cells, highly specific visualization of this cell type requires active targeting^9,10^ of ^19^F tracers. Here, we raise this approach to a new level and introduce specific and multimodal PFC targeting for systemic and non-invasive 3D mapping of neutrophil dynamics by combined in vivo ^1^H/^19^F MRI with subsequent ex vivo validation by flow cytometry and fluorescence microscopy. In a first step, we proved the feasibility of tracking neutrophil recruitment in mice and expanded this thereafter to human neutrophils. With this approach, we were not only able to target neutrophils in the circulation but also within their hematopoietic niches and to follow their migration in vivo into injured tissue over time.

## Results

### Targeting murine neutrophils via murine neutrophil-specific peptide

**Extended Data Fig. 1:**
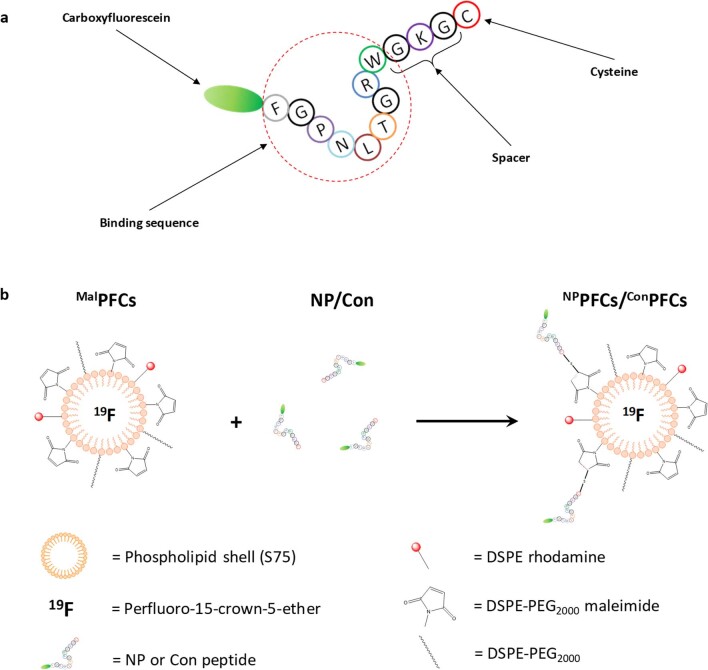
Synthesis scheme for PFC-based nanotracers to target neutrophils. **a**, Basic structure of the used peptides for targeting neutrophils. The N-terminal carboxyfluorescein is incorporated for fluorescence-based analysis. For coupling the peptides to preformed ^Mal^PFCs, a cysteine with a free sulfhydryl group is located at the C-terminus, next to the GGG spacer. The red dashed circle indicates the neutrophil binding sequence. **b**, For generating ^NP^PFCs, preformed ^Mal^PFCs were incubated with the peptides over night to form ^NP/Con^PFCs. ^Mal^PFCs were equipped with a separate label (rhodamine) to control for a potential dissociation of binding peptide and PFC. To impair the passive uptake by monocytes/macrophages poly-ethylene-glycol (PEG) coupled lipids (DSPE-PEG_2000_ = 1,2-distearoyl-sn-glycero-3-phosphoethanolamine-N-[amino(poly-ethylene glycol)-2000]) were inserted in a 5 mol% ratio into the phospholipid shell of PFCs.

**Extended Data Fig. 2:**
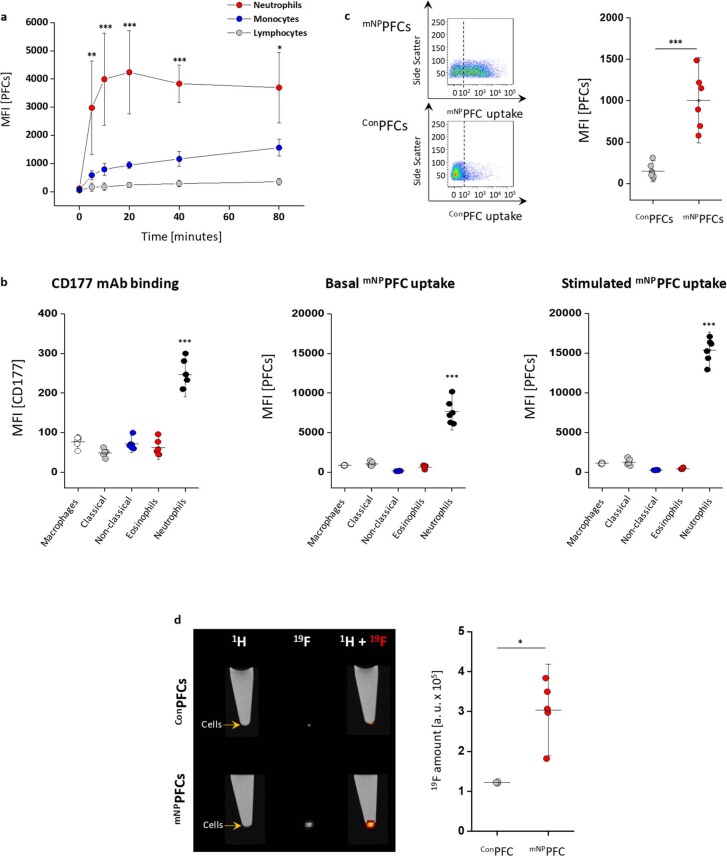
Specific targeting of neutrophils by ^mNP^PFCs. **a**, Flow cytometric analysis of murine blood leukocytes incubated with ^mNP^PFCs over 80 min illustrating their predominant uptake by neutrophils (red) as compared to monocytes (blue) and lymphocytes (gray). **b**, To further validate specificity, binding of CD177 mAb as well as incorporation of ^mNP^PFCs under basal and LPS-stimulated conditions were determined. Almost no binding/uptake into classical or non-classical monocytes as well as macrophages or eosinophils could be detected. In contrast to all other immune cell subsets, solely neutrophils showed a significant binding/uptake. **c**, Labelling of bone marrow neutrophils isolated 2 h upon intravenous injection of ^mNP^PFCs or ^Con^PFCs as determined via flow cytometry indicating a strong uptake of ^mNP^PFCs and almost no incorporation of ^Con^PFCs. **d**, To verify that cellular uptake of ^mNP^PFCs can be visualized by ^19^F MRI, we isolated murine neutrophils from the bone marrow, incubated the cells with ^mNP^PFCs or ^Con^PFCs for 3 h at 37 °C, washed intensively and pelleted the cells by centrifugation. Subsequently, cells were analyzed by ^1^H/^19^F MRI. Left: ^1^H/^19^F MRI of centrifugation tubes with neutrophils treated with ^mNP^PFCs (lower panel) or ^Con^PFCs (upper panel). The small neutrophil cell pellet is located at the bottom of the tube and can be identified by ^1^H MRI (1^st^ column) as slightly darker structure (arrows) compared to the bright phosphate buffer; corresponding ^19^F MR images (2^nd^ column) and superimposition of ^1^H/^19^F datasets (3^rd^ column, with ^19^F data color-coded in hot iron). Quantification of ^19^F signals (4^th^ column) clearly indicate substantial stronger ^19^F uptake in ^mNP^PFC-treated neutrophils (red) compared to cells incubated with ^Con^PFCs (gray); a.u. = arbitrary units. Data are means ± SD of n = 4 (a), n = 6 (b), n = 6 (c) or n = 3-5 (d) independent experiments; * = p > 0.05, ** = p < 0.01, *** = p < 0.001, verified by two-way ANOVA (a), one-way ANOVA (b) or two-sided students t-test (c, d). Source data

For targeting murine neutrophils with PFCs, we used a small peptide (murine neutrophil-specific peptide, mNP) recently identified by phage display screening to specifically bind to the neutrophil-specific receptor CD177 (ref. ^11^); a peptide with randomized sequence served as the control (Con). We modified these core peptides (Extended Data Fig. 1a) N terminally with carboxyfluorescein for fluorescence detection and C terminally with cysteine for coupling to maleimide PFCs (^Mal^PFCs) to generate ^mNP^PFCs and ^Con^PFCs, respectively. Preformed ^Mal^PFCs were equipped with a separate fluorescence label (rhodamine) for analysis of cellular uptake and to control for potential dissociation of the binding ligand and PFC (Extended Data Fig. 1b) by fluorescence-based methods. Importantly, all targeted PFCs were additionally PEGylated to block ‘passive’ uptake by phagocytic cells^12^. Subsequently, isolated murine immune cells were exposed ex vivo to the generated PFCs, and their targeting specificity was verified by flow cytometry. We observed a rapid and strong neutrophil-specific uptake of ^mNP^PFCs, whereas only minor incorporation was observed for other immune cells, which is in line with the lack of CD177 expression in these other immune cell subtypes (Extended Data Fig. 2a,b). Similar results were obtained by ^1^H/^19^F MRI (Extended Data Fig. 2d; for superimposing the images of both nuclei, a ‘hot iron’ color look-up table was applied to ^19^F images). Next, we verified the in vivo uptake of intravenously applied ^mNP^PFCs by neutrophils within the bone marrow by flow cytometry. As soon as 2 h after intravenous (i.v.) injection, we found strong labeling of bone marrow neutrophils by ^mNP^PFCs, while only negligible uptake was observed for ^Con^PFCs (Extended Data Fig. 2c).

**Extended Data Fig. 3:**
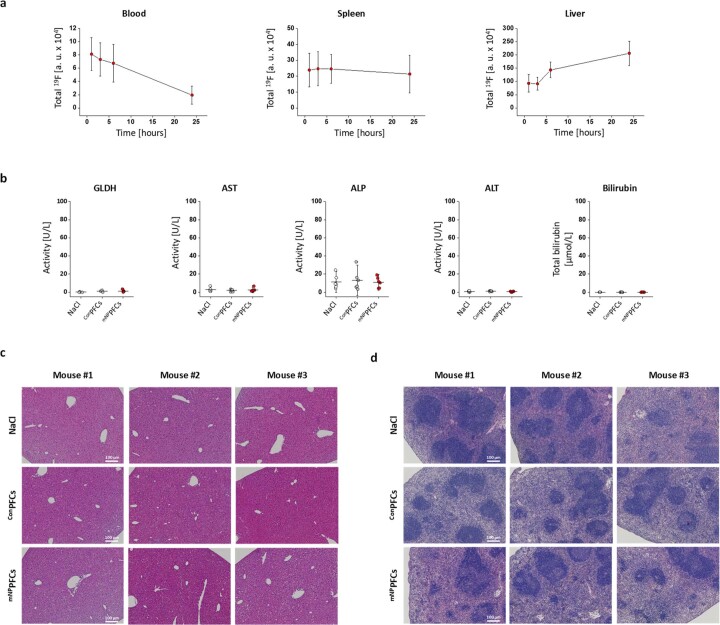
Assessment of ^mNP^PFC/^Con^PFC biodistribution, liver serum markers and histology of liver and spleen. **a**, Biodistribution of intravenously injected ^mNP^PFCs was determined *via* ^19^F MRI measurements at distinct time points after injection (1, 3, 6 and 24 h after injection). Shown are quantification of fluorine signal intensities in the blood, liver and spleen indicating a fast wash-out from the blood accompanied by a strong influx into the liver while signal intensities in the spleen were constant over the entire observation period; a.u. = arbitrary units. **b**, Serum levels of GLDH (glutamate dehydrogenase), AST (aspartate aminotransferase), ALP (alkaline phosphatase), ALT (alanine aminotransferase) and bilirubin after intravenous application of NaCl as control, ^Con^PFCs or ^mNP^PFCs. **c** + **d**, 4 µm sections of liver (C) and spleen (D) stained with hematoxylin and eosin. Organs were excised and processed for histology 24 h post injection of NaCl (upper row), ^Con^PFCs (middle), ^mNP^PFCs (lower). Representative liver/spleen images of organs from three different animals are shown. Scale bars indicate 100 µm. Data are mean values ± SD of n = 6 (a), n = 4-6 (b) and n = 6 (c + d) independent experiments. Source data

In parallel, we analyzed the biodistribution of ^mNP^PFCs by ^1^H/^19^F MRI up to 24 h after i.v. injection. During this observation period, ^mNP^PFCs were cleared from the blood pool and, as expected, concurrently accumulated also in the liver and spleen (Extended Data Fig. 3a). However, neither analysis of liver serum markers (GLDH, AST, ALP, ALT, bilirubin) nor histological examination of the liver and spleen revealed any evidence for adverse side effects of ^mNP^PFCs (Extended Data Fig. 3b,c).

### Systemic 3D mapping of neutrophil dynamics by ^1^H/^19^F MRI

**Extended Data Fig. 4:**
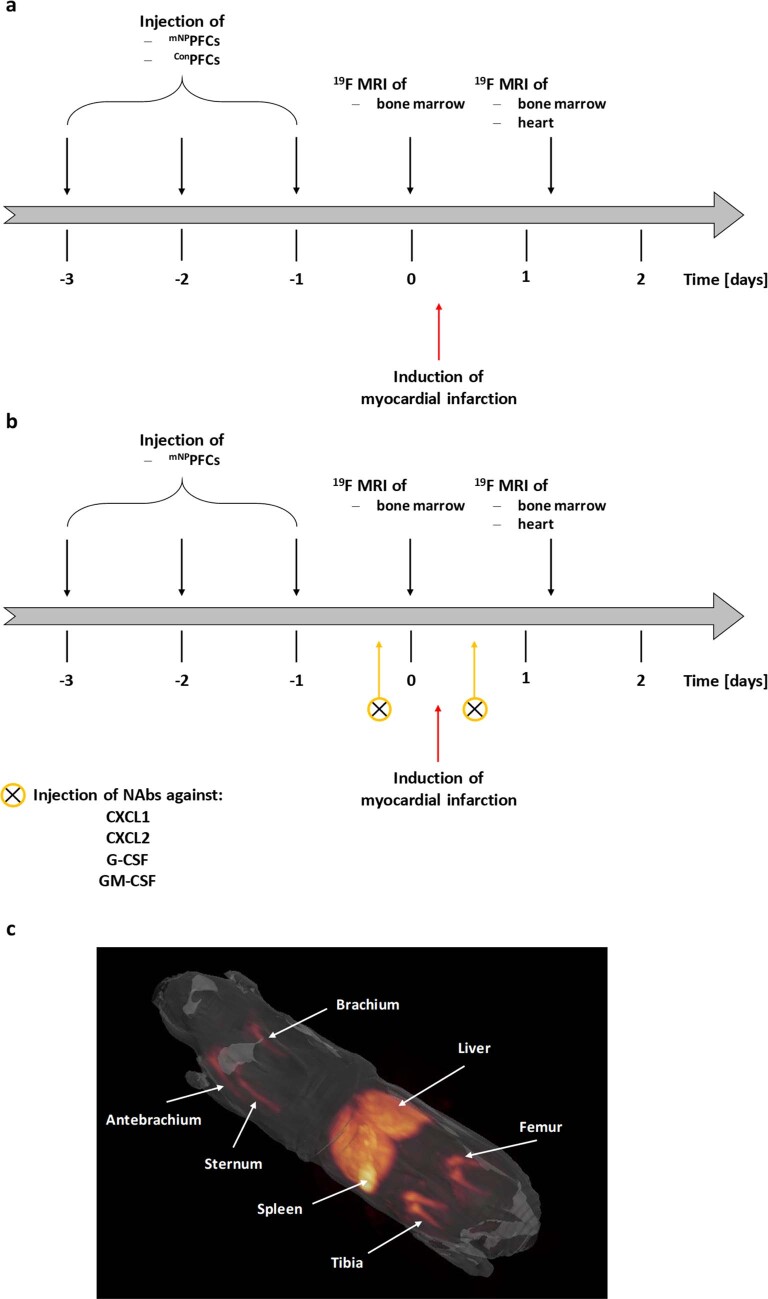
Experimental timeline and whole body ^1^H/^19^F MRI of a ^mNP^PFC-treated mouse. **a**, To label neutrophils within the bone marrow, ^mNP^PFCs (or ^Con^PFCs as control) were intravenously injected on three consecutive days (day −3, −2, −1) followed by ^1^H/^19^F MRI of the bone marrow on day 0. Subsequently, MI was induced and bone marrow and the infarcted heart were analyzed by combined ^1^H/^19^F MRI 24 h later. **b**, In separate experiments, neutralizing antibodies against CXCL1, CXCL2, G-CSF and GM-CSF were injected intraperitoneally (i. p.; 50 µg) 1 h before and 4 h post induction of MI to inhibit the MI-stimulated egress of neutrophils from the bone marrow and their infiltration into the heart. **c**, To determine the whole body biodistribution of ^mNP^PFCs, ^mNP^PFCs were intravenously injected daily over three consecutive days. Afterwards, whole body ^1^H/^19^F MRI measurements were performed to visualize the labelled neutrophils in the different bone marrow compartments. The bright ^19^F spot in the middle represents the liver – an organ known to be as major site of (PFC) nanoparticle deposition.

To follow the trafficking of neutrophils from their hematopoietic niches into inflammatory foci, we used a model of cardiac ischemia and reperfusion injury (MI), well known to be associated with acute and massive neutrophil recruitment into the injured myocardium^13^. For monitoring the fate of neutrophils upon MI, mice received daily intravenous injections of ^mNP^PFCs over 3 d before MI, and systemic labeling of neutrophils within the distinct bone marrow compartments was verified by in vivo ^1^H/^19^F MRI. Subsequently, mice were subjected to MI and, after 24 h, again scanned by MRI and/or analyzed by flow cytometry (timeline in Extended Data Fig. 4a,b).

**Fig. 1:**
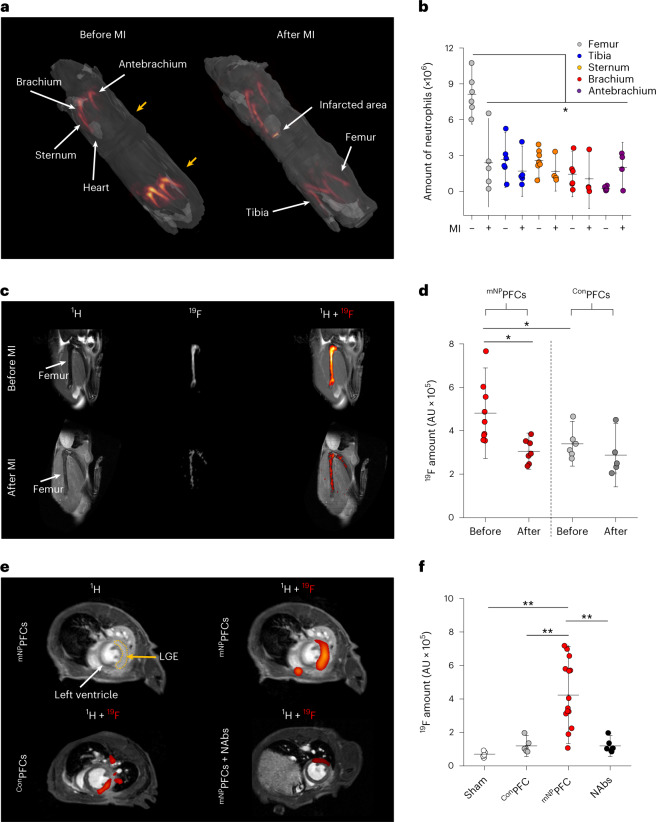
Mapping the trafficking of murine neutrophils after MI by ^mNP^PFCs in vivo. **a**, Whole-body 3D ^1^H/^19^F MRI for systemic in vivo visualization of neutrophils before and after MI. Anatomical ^1^H data were rendered transparent in grayscale with ^19^F data overlaid in orange and red; for the sake of clarity, signals from the liver and spleen were faded out. Left, intravenous application of ^mNP^PFCs before MI resulted in in situ labeling of neutrophils within their hematopoietic niches, showing the most prominent ^19^F signal in the femur and tibia. The yellow arrows indicate the areas of the local scans from the femur and heart in **c**,**e**, respectively. Right, re-investigation 24 h after MI revealed a pronounced reduction of ^19^F signals in the bone marrow of the femur and tibia with simultaneous appearance of ^19^F labeling in the infarcted heart. **b**, Post-mortem flow cytometry of the different bone marrow compartments confirmed the in vivo findings in that the femur was not only the bone containing the highest number of neutrophils before MI but also the compartment with the strongest release of neutrophils as compared to all other bones. **c**, Focal ^1^H/^19^F MR images of the bone marrow after in vivo labeling with ^mNP^PFCs and ^Con^PFCs and subsequent MI. Left, the first column displays anatomical ^1^H MR images of the femur, the second column shows corresponding background-free ^19^F MR images, and the third column is an overlay of both datasets showing strong ^19^F signals within the bone marrow after ^mNP^PFC labeling (top) and a substantial signal drop 24 h after MI (bottom). **d**, Quantification demonstrated a significant reduction in ^19^F femur signals after MI in the ^mNP^PFC-treated group, whereas application of ^Con^PFC resulted in low baseline labeling and almost no change after MI. AU, arbitrary units. **e**, Local ^1^H/^19^F MR images of the thorax revealed concomitant appearance of distinct ^19^F signals in the infarcted region when bone marrow neutrophils were labeled with ^mNP^PFCs. Top left, anatomical ^1^H MR image with delineation of the infarcted myocardium by LGE (dotted line). Top right, overlay with the corresponding ^19^F MR image confirms matching of the fluorine signal with detected LGE patterns. Bottom left and right, substantially lower ^19^F deposition within the infarcted myocardium was observed after labeling with ^Con^PFCs (left) or ^mNP^PFCs when neutrophil egress was inhibited by neutralizing antibodies (NAbs, right). Neutralizing antibodies against CXCL1, CXCL2 and granulocyte colony-stimulating factor (G-CSF) as well as granulocyte–macrophage colony-stimulating factor (GM-CSF) were intraperitoneally injected 1 h before and 4 h after induction of MI (50 µg each at both time points). **f**, Quantification of the cardiac ^19^F MR signal for all treatments. Data are mean ± s.d. of *n* = 4–7 (**b**), *n* = 5–9 (**d**) or *n* = 6–15 (**f**) independent experiments; *P < 0.05, **P < 0.01, verified by one-way ANOVA. Source data

**Extended Data Fig. 5:**
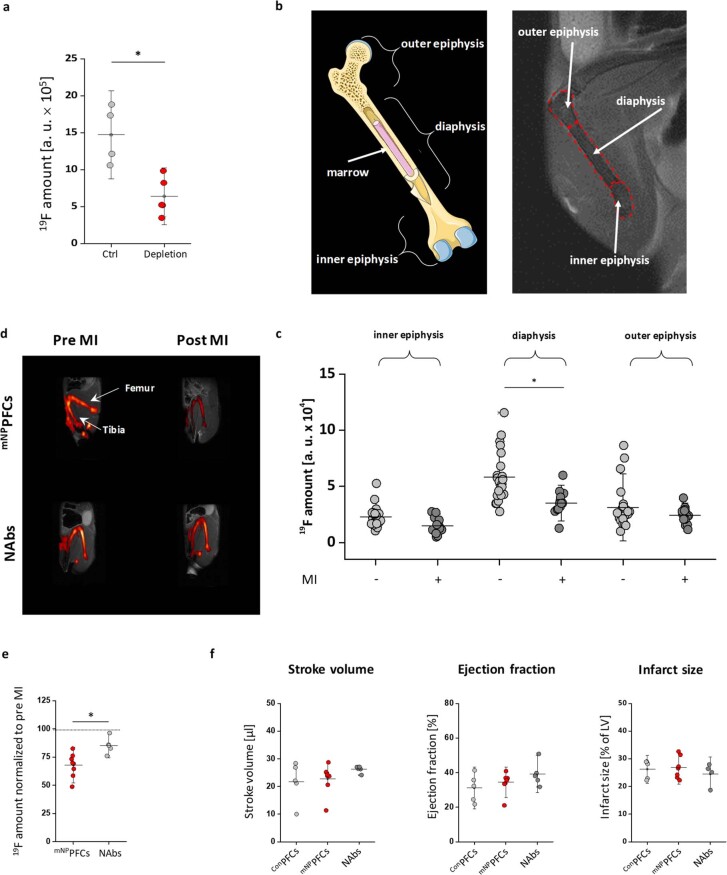
^mNP^PFC incorporation into the bone marrow and cardiac function after MI. **a**, ***Impact of neutrophil depletion on*** ^***mNP***^***PFC incorporation into the bone marrow:*** For *in vivo* labeling of bone marrow neutrophils, ^mNP^PFCs were injected intravenously and 24 h later the ^19^F signal within the femur and tibia was determined (gray). Depletion of neutrophils by Ly6G Ab injection 48 and 24 h prior ^mNP^PFC injection lead to significantly decreased ^19^F bone marrow signals (red). **b** + **c**, ***Locoregional analysis of the femur*** ^***19***^***F signal before and after MI:*** **b**, Left: Schematic overview of the femur with the compartments inner/outer epiphysis and diaphysis. The marrow mainly belongs to the diaphysis whereas the growth of the bones takes place at the inner and outer epiphysis. At the right, an exemplarily compartmentalization of a murine ^1^H MR scan of the femur with the inner and outer epiphysis as well as the diaphysis is shown. **c**, Quantification of the ^19^F signal in the different femur compartments revealed highest ^mNP^PFC uptake in the diaphysis before MI and a significant drop in this compartment after MI. The inner and outer epiphysis are characterized by lower fluorine intensities prior MI and smaller, non-significant decreases upon MI. **d** + **e**, ***Retention of bone marrow neutrophils by NAbs after MI:*** **d**, Antibodies against CXCL1, CXCL2, G-CSF and GM-CSF were injected intraperitoneally (i. p.) 1 h before and 4 h post induction of MI to inhibit the MI-stimulated egress of neutrophils from the bone marrow and their infiltration into the heart. Left: Combined ^1^H/^19^F MRI of the bone marrow (BM) before (left) and after MI (right). **e**, For quantification, the BM ^19^F signal post MI was normalized to the ^19^F signals detected before induction of MI. **f**, ***Ischemic area and functional cardiac parameters 1 day post MI:*** To exclude any effects on PFC distribution caused by differences in myocardial tissue impairment after MI between the individual experimental groups, we determined ischemic area, stroke volume and ejection fraction from ^1^H MRI LGE cine measurements for each condition (LV = left ventricle). However, no significant differences between ^Con^PFCs-, ^mNP^PFCs- or ^mNP^PFCs + NAbs-treated groups were found. Data are means ± SD of n = 4-5 (a), n = 7 – 11 (c), n = 5 – 8 (e) and n = 5 – 8 (f) independent experiments; * = p < 0.05, verified by two-sided students t-test (a, e) or one-way ANOVA (c). Source data

Whole-body ^1^H/^19^F MRI before MI corroborated the finding that the labeling protocol with ^mNP^PFCs resulted in strong ^19^F uptake by bone marrow neutrophils, particularly in the femur, tibia, brachium, antebrachium and sternum, with the highest ^19^F signals originating from the femur and tibia (Fig. 1a, left; for the sake of clarity, ^19^F signals from the liver and spleen have been faded out (see Extended Data Fig. 4c for the enclosure of these organs)). Re-investigation 24 h after induction of MI revealed substantial reduction of ^19^F signals in the bone marrow of the femur and tibia (Fig. 1a, right) with concomitant appearance of ^19^F labeling in the infarcted heart. In line with these in vivo findings, flow cytometry identified the femur as the main neutrophil reservoir and also as the bone marrow compartment with the largest decrease in neutrophils 24 h after MI (Fig. 1b). Because this indicated the femur as the most important source for neutrophil release upon MI, we focused for the following on a more localized mapping approach with optimized spatial resolution and sensitivity for ^1^H/^19^F MRI of the hindlimb and heart. Of note, independent experiments with anti-Ly6G antibodies applied 48 and 24 h before ^mNP^PFC application to deplete neutrophils resulted in strongly decreased ^19^F signals in the bone marrow (Extended Data Fig. 5a), further corroborating the idea that these signals are predominantly caused by ^mNP^PFC labeling of neutrophils.

**Fig. 2:**
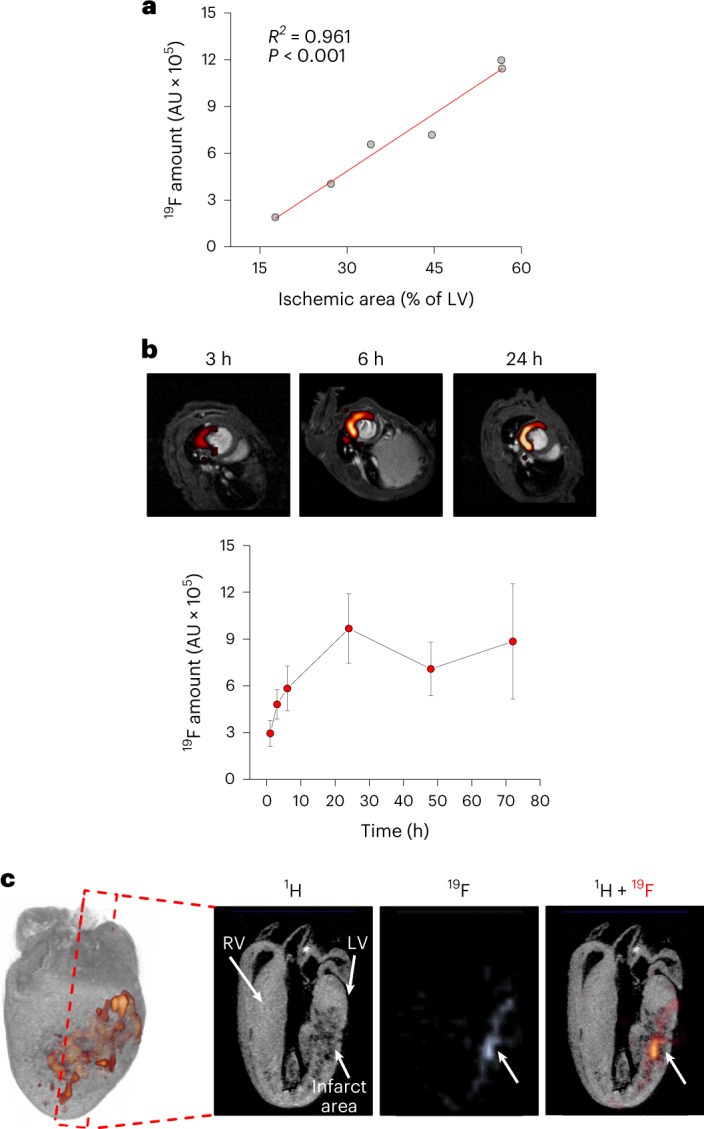
Correlation of the ^19^F signal with ischemic area, its temporal development and ex vivo validation. **a**, Linear regression between ischemic area (LGE) and fluorine signal within the infarcted myocardium. **b**, Time course of neutrophil infiltration into the injured heart. The fluorine signal was determined 1, 3, 6, 24, 48 and 72 h after induction of MI (examples are given at the top). **c**, To further corroborate the location of the ^19^F signal within the infarcted myocardium, hearts were excised, fixed with paraformaldehyde and analyzed by ex vivo high-resolution ^1^H/^19^F 3D MRI. In long-axis ^1^H MR images, the infarcted area can be unequivocally identified as a dark structure within the bright intact myocardium. Importantly, the corresponding ^19^F signal is restricted to the infarcted area, indicating infiltration of ^mNP^PFC-labeled neutrophils only into the injured myocardium. Left, 3D volume rendering of the heart with superimposed ^19^F signal. LV, left ventricle; RV, right ventricle. Data are mean ± s.d. of *n* = 6 (**a**) and *n* = 5–6 (**b**) independent experiments. Source data

**Fig. 3:**
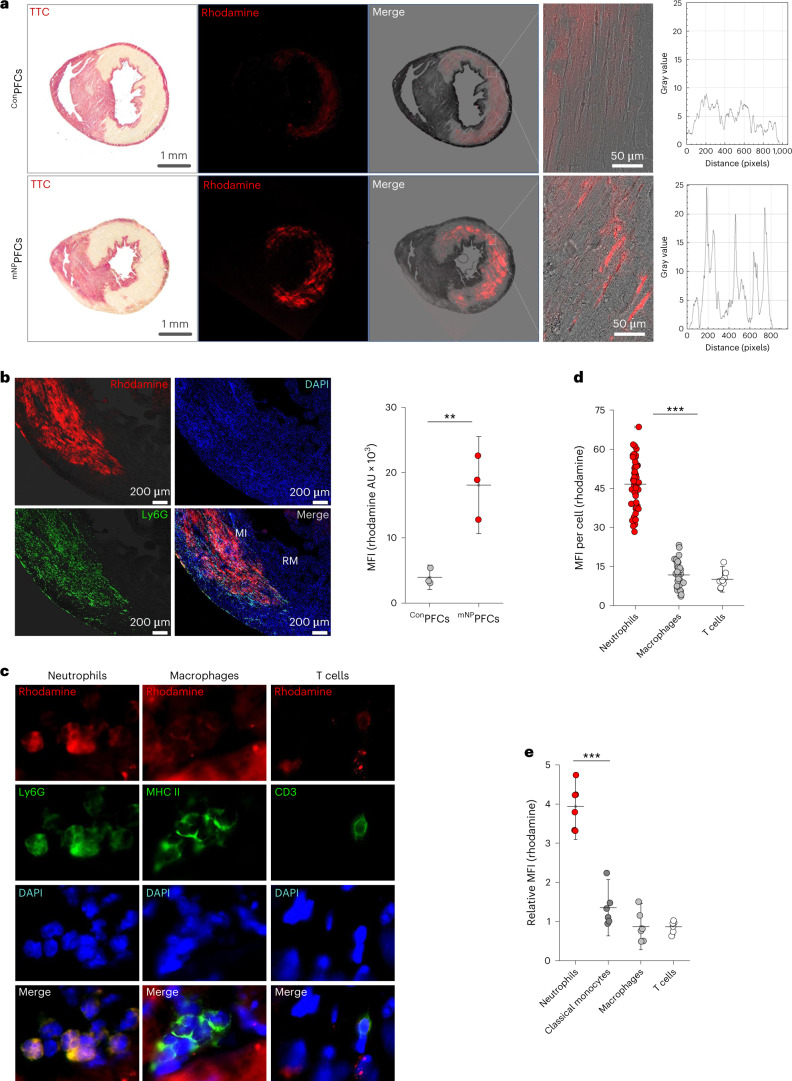
Identification of ^mNP^PFC-labeled neutrophils in the infarcted heart. **a**, For identification of ^mNP^PFC-labeled neutrophils in the infarcted heart, mice received injections of rhodamine-tagged ^mNP^PFCs or ^Con^PFCs 2 h before induction of MI and hearts were excised 2 h after induction of MI. The infarct area was visualized by 2,3,5-triphenyltetrazolium chloride (TTC) staining, which was found to colocalize with strong rhodamine signals derived from ^mNP^PFC-labeled cells (bottom). By contrast, ^Con^PFCs led to much weaker and spread-out signals only (top). Magnifications and histograms (fourth and fifth columns) demonstrated distinct signals for ^mNP^PFCs but only diffuse patterns for ^Con^PFCs. **b**, Left, examination of infarct (MI) and remote (RM) regions revealed strong rhodamine labeling colocalized with Ly6G staining in the injured tissue. Right, quantification confirmed the selective uptake of ^mNP^PFCs versus ^Con^PFCs. **c**, Analysis of ^mNP^PFC uptake by cardiac neutrophils (left, Ly6G staining), macrophages (middle, major histocompatibility complex (MHC) II staining) and T cells (right, CD3 staining). Rhodamine signals were colocalized with neutrophils, while macrophages and T cells showed little or no signals. **d**, Quantification of mean fluorescence intensities (MFI) of individual cell types demonstrated significantly stronger labeling of neutrophils than of macrophages and T cells. **e**, To further corroborate the histological data, immune cells were isolated from the infarcted heart by Langendorff digestion and analyzed by flow cytometry. To identify the different immune cell clusters, cells were stained for CD45, CD11b, Ly6C and Ly6G. Neutrophils (CD45^+^CD11b^+^Ly6C^−^Ly6G^+^) were characterized by strong labeling after ^mNP^PFC injection, while classical monocytes (CD45^+^CD11b^+^Ly6C^+^Ly6G^−^), macrophages (CD45^+^CD11b^+^Ly6C^−^Ly6G^−^) and lymphocytes (CD45^+^CD11b^−^Ly6C^−^Ly6G^−^) exhibited only low signal intensities in relation to animals injected with ^Con^PFC. Data are mean ± s.d. of *n* = 3 (**a**), *n* = 3 (**b**), *n* = 3 (**c**), *n* = 8–48 (**d**) and *n* = 6 (**e**) independent experiments (**a**–**c**,**e**) or individual cell MFI measurements from three independent experiments (**d**). **P < 0.01, ***P < 0.001, verified by two-sided Student’s *t*-test (**b**) or one-way ANOVA. Source data

Focal scanning of the thighs before and 24 h after MI confirmed the strong decrease of ^19^F signals in the bone marrow of the femur after MI (Fig. 1c, top and bottom), which was most pronounced in the diaphysis (Extended Data Fig. 5b,c). In parallel, well-resolved images of the thorax unequivocally corroborated the simultaneous appearance of ^19^F labeling in the heart (Fig. 1e, top). Cine MRI in combination with late gadolinium enhancement (LGE) demonstrated that the detected ^19^F pattern perfectly matched the LGE-delineated myocardium (Fig. 1e, top). Quantification of ^19^F data showed the emerging ^19^F signal in the heart to be on the same order of magnitude as the decline in the bone marrow (Fig. 1d,f), strongly indicating that this is caused by ^mNP^PFC-loaded neutrophils released from the femur and entering the infarcted myocardium. Importantly, animals that received ^Con^PFCs exhibited significantly less labeling of the bone marrow before MI, which was unchanged after MI and led to only minor amounts of ^19^F labeling in the infarcted region (Fig. 1d,f). Furthermore, application of neutralizing antibodies to inhibit the egress of ^mNP^PFC-loaded neutrophils from the femur into the blood blunted MI-induced effects in the heart and femur (Fig. 1e,f and Extended Data Fig. 5d,e). Similarly, sham-operated animals treated with ^mNP^PFCs showed only negligible ^19^F signals (Fig. 1f): ischemic area (LGE) and functional impairment at this early point in time after MI were similar in all groups (Extended Data Fig. 5f). Remarkably, linear regression of the LGE-delineated myocardium and the ^19^F integral resulted in a significant correlation of ischemic area and infiltrated neutrophils 24 h after MI (Fig. 2a, adjusted *R*^*2*^ = 0.961). When extending the time window of our analysis to 1, 3, 6, 24, 48 and 72 h after MI, we observed a continuous increase in ^19^F signal up to 24 h (Fig. 2b), which is in line with the infiltration kinetics of neutrophils after MI reported in the literature^14^. To further validate that ^19^F patterns detected in vivo are localized within the infarcted myocardium, hearts were excised and analyzed by high-resolution ^1^H/^19^F MRI, which unequivocally pinpointed the ^19^F signal within the infarct area (Fig. 2c). Additional histology confirmed the specific uptake of ^mNP^PFCs by neutrophils in the infarcted heart, while signals from macrophages and monocytes were negligible (Fig. 3a–d). This was also corroborated by flow cytometry of immune cells isolated from the infarcted heart (Fig. 3e).

### Conjugation is required for specific uptake of mNP

**Fig. 4:**
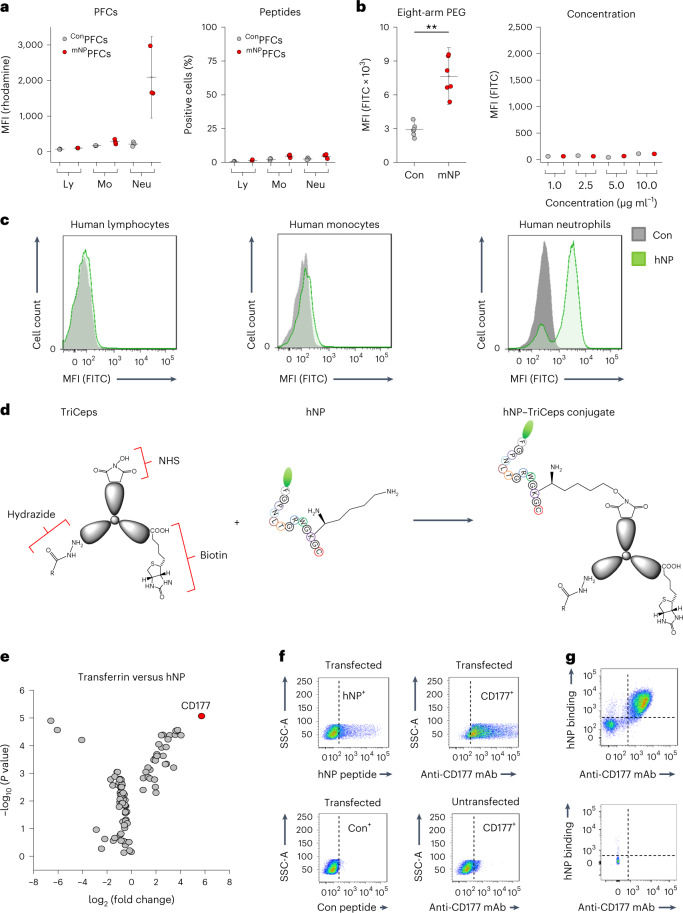
Limitations of mNP and identification of CD177 as a target for hNP on human neutrophils. **a**, Uptake of either ^mNP^PFCs and ^Con^PFCs (left) or the free peptides mNP and Con (right) by murine immune cells, quantified by flow cytometry. Note that ^mNP^PFCs (red, left) were strongly taken up by neutrophils (Neu), whereas unconjugated free mNP (red, right) did not label neutrophils. Ly, lymphocytes; Mo; monocytes. **b**, Left, mNP (red) as well as the control peptide Con (gray) was conjugated to eight-arm PEG_2000_-maleimide, and the uptake of these conjugates by neutrophils was analyzed by flow cytometry. Right, impact of increasing mNP and Con concentrations (1, 2.5, 5 and 10 µg ml^−1^) on labeling of neutrophils. **c**, Representative flow cytometry histograms of human immune cells incubated with hNP (green) or its control peptide (Con, gray). **d**, Concept for identification of the binding target of hNP using TriCeps, which consists of an *n*-hydroxysuccinimide (NHS) group for conjugation to hNP, a hydrazide for binding to different sugar structures on the cell surface and biotin for purification. **e**, Isolated human neutrophils were incubated with the hNP–TriCeps conjugate, and, thereafter, cells were lysed and subjected to affinity purification. Volcano plot of mass spectrometric analysis identified CD177 as the most likely candidate target for hNP. **f**, To confirm CD177 as the binding partner for hNP, CHO cells were transiently transfected with plasmids encoding human CD177. The binding of hNP (top left) and Con (bottom left) as well as a CD177 monoclonal antibody (mAb) (top right) was determined by flow cytometry. Untransfected cells served as the control (bottom right). SSC, side scatter. **g**, Human neutrophils were co-stained with either hNP (top) or hNP and anti-CD177 monoclonal antibody (bottom), followed by flow cytometry: a CD177-positive donor (top) and a CD177-negative donor (bottom). Data are mean ± s.d. of *n* = 5–6 (**a**) or *n* = 5–7 (**b**) independent experiments; ***P < 0.001, verified by two-sided Student’s *t*-test. Source data

**Extended Data Fig. 6:**
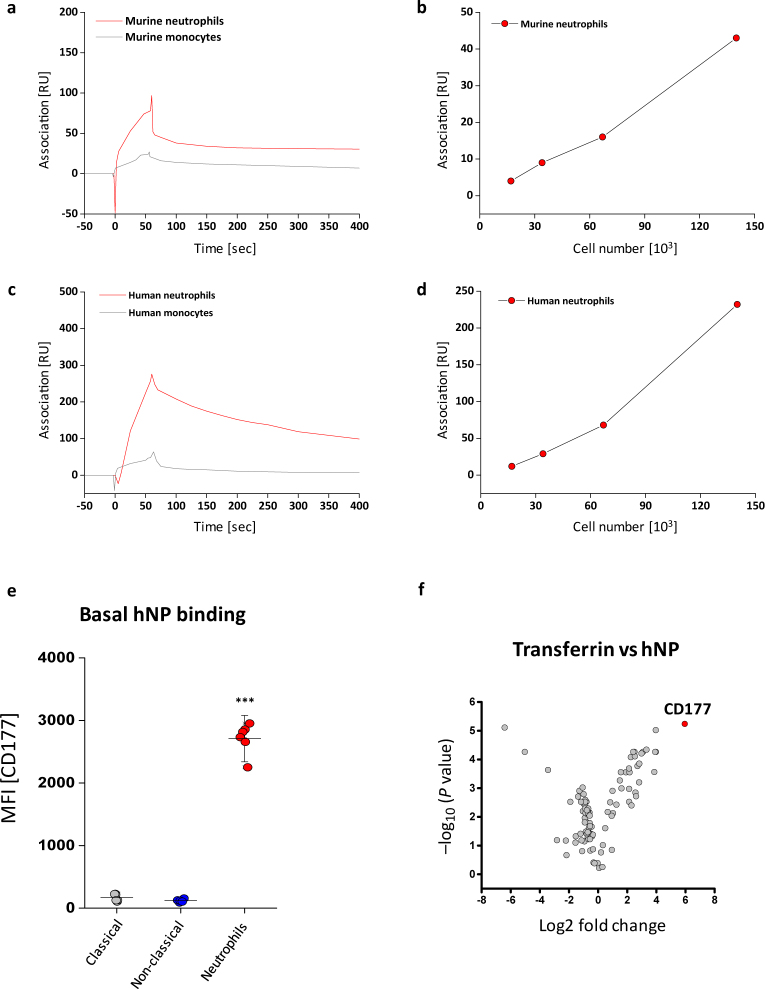
Binding characterics of human and murine NP. **a-d**, ***Surface plasmon resonance spectroscopy (SPR) of NP binding to murine/human neutrophils and monocytes:*** Representative SPR sensograms of murine **a**, and human **c**, neutrophils (red) and monocytes (gray) binding to mNP and hNP, respectively. In each case 1.4×10^5^ cells were flushed over the sensor chip. Binding curves of human and mouse neutrophils reveal a rapid association to the immobilized NP peptide and a slow dissociation rate (human K_d_: 0.00449 s^−1^, mouse K_d_: 0.00901 s^−1^). In contrast, human and mouse monocytes showed only a minor association with immobilized NP peptides. Furthermore, the SPR signal was determined for binding of the NP peptide with increasing cell numbers (1.7×10^4^, 3.4×10^4^, 6.7×10^4^, and 1.4×10^5^). Here, the observed SPR signal correlated almost linearly with the injected numbers of both murine **b**, and human **d**, neutrophils, and binding could be detected even for low cell numbers as 1.7×10^4^ neutrophils. **e**, ***Binding of hNP to human monocyte subsets and neutrophils:*** Binding of the hNP peptide as determined by flow cytometry. We detected a strong binding by human neutrophils while classical as well as non-classical monocytes showed no binding at all. **f**, ***Validating identification of the hNP surface receptor with a modified spacer sequence:*** The modified spacer contained a lysine within the glycine spacer (-GKG- instead of –GGG-) which introduces a second conjugation site for the NHS group of the TriCeps^TM^ to rule out any influence on the binding of the peptide. Subsequent mass spectroscopy revealed a similar protein profile and identified again CD177 as the most likely binding candidate. Data are means ± SD of n = 1 (a – d), n = 5-6 (e) and n = 1 (f) independent experiments; *** = p < 0.001, verified by one-way ANOVA. Source data

As described above, mNP-decorated PFCs specifically labeled murine neutrophils (Extended Data Fig. 2a,b and Fig. 4a, left), but when we additionally characterized the properties of the free mNP peptide itself, we surprisingly found that unbound mNP did not label neutrophils (Fig. 4a, right). To verify whether conjugation affects its binding, mNP was coupled to an eight-arm PEG_2000_-maleimide molecule. Subsequent exposure of murine neutrophils to these conjugates indeed revealed much stronger uptake (Fig. 4b, left). This was not related to high avidity due to enhanced local density of mNP, because, even at very high concentrations, free mNP was not taken up by murine neutrophils (Fig. 4b, right). For additional information on the binding characteristics of mNP, we performed surface plasmon resonance spectroscopy (SPR, Extended Data Fig. 6a,b). To this end, mNP was conjugated to a sensor chip, and subsequently neutrophils (red) as well as monocytes (gray) were flushed over the sensor surface. As can be clearly recognized, SPR sensorgrams revealed rapid association of neutrophils with the immobilized mNP and a slow dissociation rate. By contrast, monocytes showed only a minor association with immobilized mNP (Extended Data Fig. 6a). Altogether, these data indicate that a certain degree of conjugation of mNP is required for binding and subsequent labeling of murine neutrophils, which clearly hampers broader translational application by coupling mNP to small molecular positron emission tomography (PET) or Gd tracers.

### Targeting human neutrophils via hNP

Due to these restrictions of mNP binding, we altered the targeting strategy for human neutrophils and used a peptide that has been identified as specific for human neutrophils and does not require coupling to scaffolds for targeting (human neutrophil-specific peptide, hNP)^15^. We modified hNP as detailed above and verified its uptake by isolated human immune cells. As shown in Fig. 4c and Extended Data Fig. 6e, flow cytometry confirmed specificity of hNP for neutrophils again compared to a randomized control peptide and to other immune cell populations.

Because the binding target for hNP on human neutrophils was not yet identified, we next aimed to characterize its membrane receptor. For this, we cross-linked an hNP–TriCeps conjugate to the surface of isolated human neutrophils (Fig. 4d). After cell lysis, ligands were enriched, purified and identified by mass spectrometry^16^. In parallel, a transferrin–TriCeps conjugate was used for exclusion of nonspecific binding candidates. Subsequent data analysis revealed enrichment of 24 proteins for hNP compared to transferrin (Supplemental Table 1). Remarkably, volcano plots identified human CD177 as the most abundant protein (Fig. 4e), indicating that it is a binding partner for hNP, analogous to mNP binding to murine CD177. This was further corroborated by a second set of experiments with a modified spacer (Extended Data Fig. 6f). Subsequently, we transiently transfected Chinese hamster ovary (CHO) cells with plasmids encoding human CD177 and confirmed that transfected cells bound hNP but not the control peptide (Fig. 4f). Only the high-affinity anti-CD177 monoclonal antibody as a positive control displayed as strong an effect as hNP; untransfected CHO cells did not bind hNP or monoclonal antibodies against CD177, indicating absence of endogenous CD177 expression (Fig. 4f). Additionally, we stained neutrophils with hNP only or in combination with monoclonal antibodies against CD177, demonstrating that only CD177-positive neutrophils co-stain with hNP and anti-CD177 monoclonal antibody (Fig. 4g, top). Interestingly, a fraction of the human population does not express CD177 on neutrophils, and staining blood cells from CD177-negative volunteers did not show binding of anti-CD177 monoclonal antibody or hNP (Fig. 4g, bottom). Analogous to the murine neutrophil peptide, we finally performed SPR analyses of hNP: here, neutrophils exhibited an even stronger initial binding phase and slow dissociation, while monocytes displayed only negligible binding to the sensor chip (Extended Data Fig. 6c,d).

**Extended Data Fig. 7:**
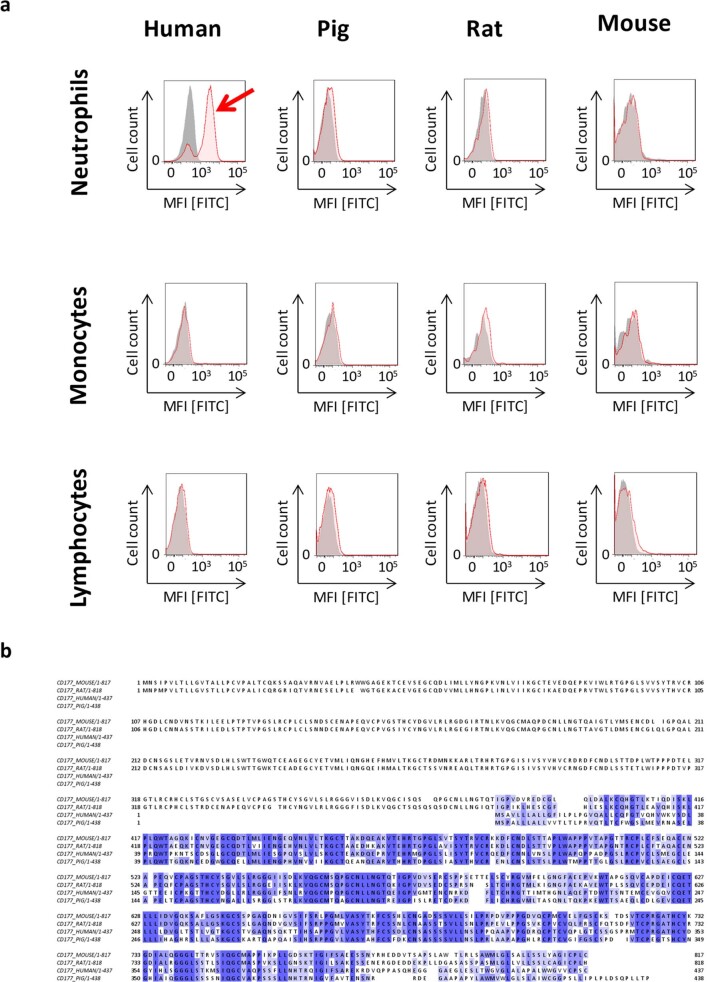
Cross-species analysis of hNP binding and CD177 amino acid sequence. **a**, ***hNP does not bind to neutrophils from pigs, rats or mice:*** Determination of cross-species reactivity of hNP for pig, rat and mouse immune cells (neutrophils, lymphocytes, monocytes) analyzed by flow cytometry. The histogram overlays show binding studies with human immune cells (1^st^ column) and leukocytes isolated from the blood of pigs (2^nd^ column), rats (3^rd^ column) or mice (4^th^ column); gray = untreated cells, red = hNP treated cells. **b**, ***Cross-species analysis of CD177 amino acid sequence:*** Alignment of the protein sequence of CD177 from humans, mice, rats and pigs. Dark blue shows highly conserved sequence areas between all four species. Light blue indicates less conserved amino acids and white represents non-conserved areas.

Of note, we observed no uptake of hNP by neutrophils from pigs, rats or mice (Extended Data Fig. 7a). Sequence analysis revealed only low-grade amino acid conservation of CD177 between those species, which may account for the specificity of hNP for human neutrophils (Extended Data Fig. 7b).

### Visualization of human neutrophils by ^19^F MRI using ^hNP^PFCs

**Fig. 5:**
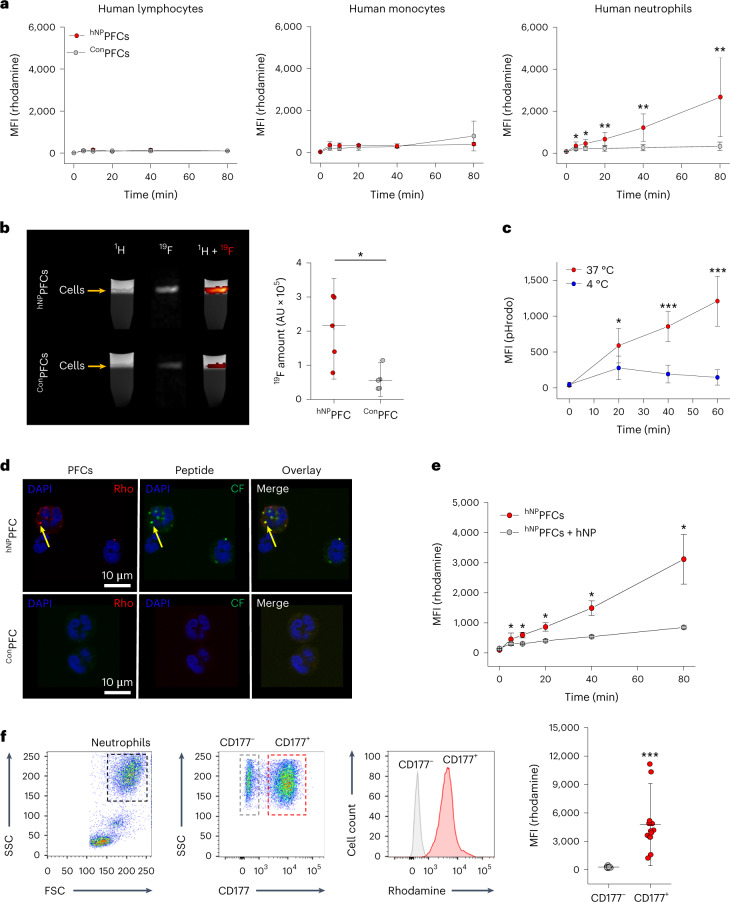
Specific targeting of human neutrophils by ^hNP^PFCs. **a**, Uptake of ^hNP^PFCs (red) or ^Con^PFCs (gray) by human lymphocytes, monocytes or neutrophils over time as determined by flow cytometry. **P < 0.01. **b**, For MRI analysis, human immune cells were incubated with ^hNP^PFCs (top row) or ^Con^PFCs (bottom row). After several washing steps, cells were purified by density gradient centrifugation and analyzed by MRI. First column, ^1^H MR image of the centrifugation tube with the cell layer (arrow) on top of the dark Percoll layer; second column, ^19^F MR image of the same area; third column, merge of both datasets. Quantification of the ^19^F data is shown on the right. **c**, hNP was conjugated to the pH-sensitive dye pHrodo, incubated with neutrophils at 4 °C (blue) and 37 °C (red) and analyzed by flow cytometry. **d**, Confocal microscopy of neutrophils incubated with ^hNP^PFCs (top) or ^Con^PFCs (bottom). Fluorescence signals of PFCs (rhodamine; Rho) as well as ligands (carboxyfluorescein; CF) were recorded, and nuclei were counterstained with 4′,6-diamidino-2-phenylindole (DAPI) (blue). Accumulation of ^hNP^PFCs within the endosomal–lysosomal system is highlighted by yellow arrows. **e**, Neutrophils were pretreated with hNP at 4 °C (gray) or left untreated (red), followed by incubation with ^hNP^PFCs. At distinct time points, uptake of ^hNP^PFCs was determined by flow cytometry. **f**, Human blood immune cells were incubated with ^hNP^PFCs followed by staining for CD177 and flow cytometry. Neutrophils were gated with the appropriate FSC and SSC settings, and both subpopulations (CD177^−^, CD177^+^) were identified by CD177 staining. For the histogram plot and quantification, while CD177^+^ neutrophils were positive for the rhodamine label of the ^hNP^PFCs (red), CD177^−^ neutrophils were rhodamine negative (gray). FSC, forward scatter. Data are mean ± s.d. of *n* = 4–6 (**a**), *n* = 5–6 (**b**), *n* = 5–6 (**c**), *n* = 3 (**d**), *n* = 3–4 (**e**) and *n* = 13 (**f**) independent experiments; *P < 0.05, ***P < 0.001, verified by two-way ANOVA (**a**,**c**,**e**) or two-sided Student’s *t*-test (**b**). Source data

**Extended Data Fig. 8:**
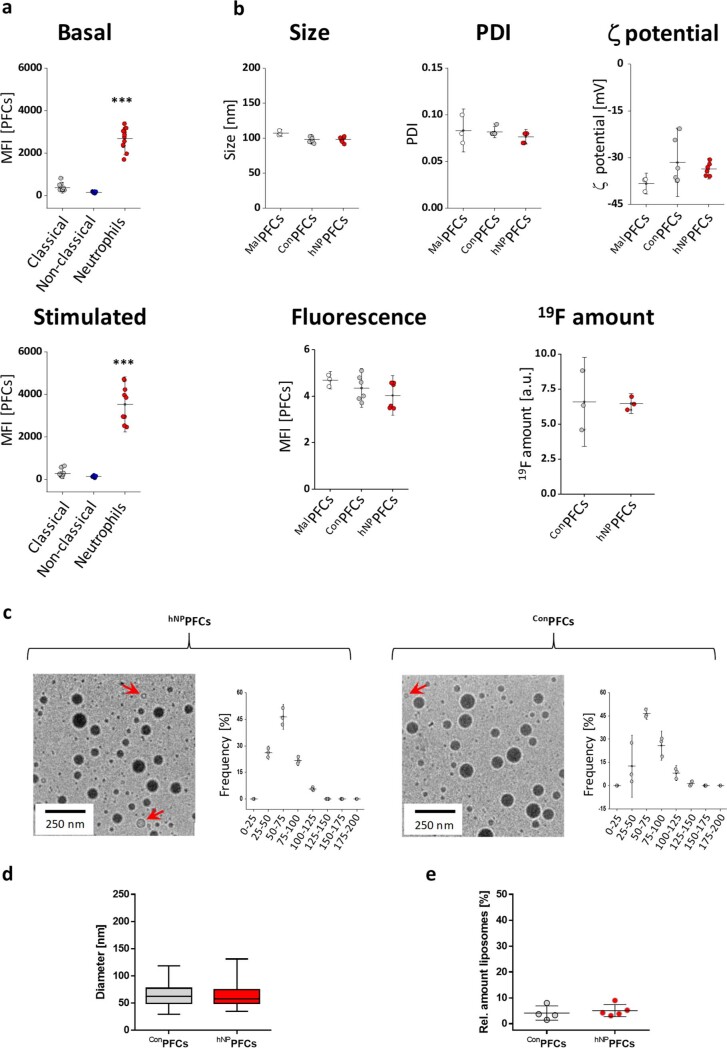
Physicochemical properties and cellular uptake of ^hNP^PFCs. **a**, ***Cellular uptake of*** ^***hNP***^***PFCs by human monocyte subsets and neutrophils:*** Human immune cells isolated from the blood of healthy volunteers were incubated with ^hNP^PFCs. Subsequently, cells were stained with CD11b, CD14 and CD16 to differentiate between neutrophils (red), classical monocytes (gray) and non-classical monocytes (blue) and the cells were analyzed by flow cytometry. The cellular uptake of ^hNP^PFCs was determined under unstimulated conditions (top) and after treatment with 1 µg/ml LPS (bottom). **b**, ***Physicochemical properties of the generated nanotracers:*** Characterization of size, size distribution and ζ potential of ^Mal^PFCs and targeting PFCs (^hNP^PFCs, ^Con^PFCs) by dynamic light scattering (DLS). The rhodamine fluorescence signal of the PCS was determined by IVIS imaging and the ^19^F content by ^19^F MRI. **c-e**, ***Cryo transmission electron microscopy of the generated nanotracers:*** **c**, Cryo transmission electron microscopy (cryo-TEM) of ^hNP^PFCs and ^Con^PFCs. Graphs: The diameter of the PFCs was determined manually from three images categorized into 25 nm groups. The relative frequency of the number of PFCs in the range of 0-200 nm is displayed. Data are derived from three images for ^hNP^PFCs and ^Con^PFCs each. **d**, Box plot (box and whiskers; min to max) of the diameter of ^Con^PFCs/^hNP^PFCs calculated from cryo-TEM images. **e**, Relative amount of liposomes (%) in individual cryo-TEM images. The number of PFCs and liposomes was manually determined (n = 4/5 images). Red arrows in the cryo-TEM images indicate liposomes. Data are mean values ± SD of n = 5-6 (a), n = 3-6 (b), n = 3 (c), n = 3 (d) and n = 6 (e) independent experiments; *** = p < 0.001, verified by one-way ANOVA (a). Source data

Next, hNP was coupled to PFCs, and the formed ^hNP^PFCs were evaluated for labeling of immune cells from human blood. ^hNP^PFCs avidly bound to neutrophils, whereas ^Con^PFCs displayed only marginal binding (Fig. 5a). Importantly, we observed no binding to lymphocytes and only minor uptake by monocytes and their subtypes even under lipopolysaccharide (LPS) stimulation (Extended Data Fig. 8a). To confirm that the hNP-based targeting approach is also suitable for ^19^F MRI, human neutrophils were incubated with ^hNP^PFCs, separated from free ^hNP^PFCs by density gradient centrifugation and subsequently subjected to combined ^1^H/^19^F MRI. In *T*_2_-weighted ^1^H magnetic resonance (MR) images, cells can be identified as a small dark layer within the ‘light’ buffer band (arrow) superimposed on the dark Percoll layer below (Fig. 5b). As can be clearly recognized, cells exposed to ^hNP^PFCs displayed significantly stronger ^19^F signals than controls.

### ^hNP^PFC uptake and impact on human neutrophil function

For longitudinal tracking, it would be highly desirable that ^hNP^PFCs not only bind to but are also internalized by neutrophils to avoid shearing off of the targeting moieties from the cell surface. To explore uptake of the targeting peptide upon binding, we coupled the pH-sensitive dye pHrodo to hNP (^rodo^hNP). Incubation of neutrophils with ^rodo^hNP at 37 °C, but not at 4 °C, led to a massive increase in fluorescence intensity (Fig. 5c), strongly indicating energy-dependent internalization of the peptide and its deposition within the acidic endosomal–lysosomal compartments. Next, we monitored uptake of the coupled ^hNP^PFCs by confocal immunofluorescence microscopy and observed unambiguous co-staining (yellow arrows) of rhodamine (→PFC) and carboxyfluorescein (→hNP) within cells, while ^Con^PFCs resulted only in background signals (Fig. 5d).

In a competition approach, neutrophils were pretreated with hNP at high concentrations to block CD177-binding sites, which strongly inhibited subsequent ^hNP^PFC uptake (Fig. 5e), further corroborating hNP specificity and excluding passive endocytosis. Moreover, differentiation between CD177^−^ and CD177^+^ neutrophils demonstrated that only the latter population could be labeled with ^hNP^PFCs (Fig. 5f). Detailed physicochemical characterization of the emulsions excluded the idea that targeting of ^hNP^PFCs may be related to any differences in size, size distribution, *ζ* potential, fluorescence intensity or ^19^F content (Extended Data Fig. 8b). Furthermore, only tiny amounts of empty liposomes were observed as undesired side products of the PFC preparation (Extended Data Fig. 8c–e, red arrows).

**Fig. 6:**
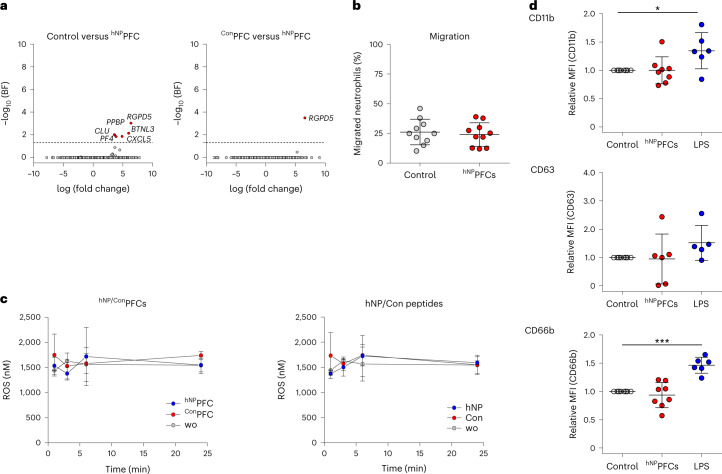
Targeting with ^hNP^PFCs does not impact human neutrophil properties. **a**, Differentially expressed genes identified by bulk RNA sequencing of human blood neutrophils after incubation with ^hNP^PFCs, ^Con^PFCs or NaCl as the control. Volcano plots of differentially expressed genes for human neutrophils treated with saline compared to ^hNP^PFCs (left) or ^Con^PFCs compared to ^hNP^PFCs (right). Genes marked in red are significantly upregulated with a log_2_ (fold change) greater than 1.5. BF, Bonferroni-corrected *P* values of the false discovery rate. In total, 45,413 RNA transcripts were analyzed. **b**, Migration of neutrophils treated with ^hNP^PFCs (red) or left untreated (gray) toward IL-8. **c**, Neutrophils were incubated with ^hNP^PFC or ^Con^PFC or the hNP or Con peptides, and ROS generation was determined by enzyme-linked immunosorbent assay. wo, without any PFC. **d**, Cell surface expression of neutrophil activation markers: neutrophils were left untreated (gray, control), incubated with ^hNP^PFCs (red) or stimulated with LPS (blue) as the positive control, followed by flow cytometry for CD11b (top), CD63 (middle) or CD66b (bottom). Mean fluorescence intensities were normalized to those of untreated cells. Data are mean ± s.d. of *n* = 4 (**a**), *n* = 9–10 (**b**), *n* = 5–6 (**c**) and *n* = 5–8 (**d**) independent experiments; *P < 0.05, ***P < 0.001, verified by two-sided Bonferroni-corrected ANOVA (**a**) or two-way ANOVA (**d**). Source data

**Extended Data Fig. 9:**
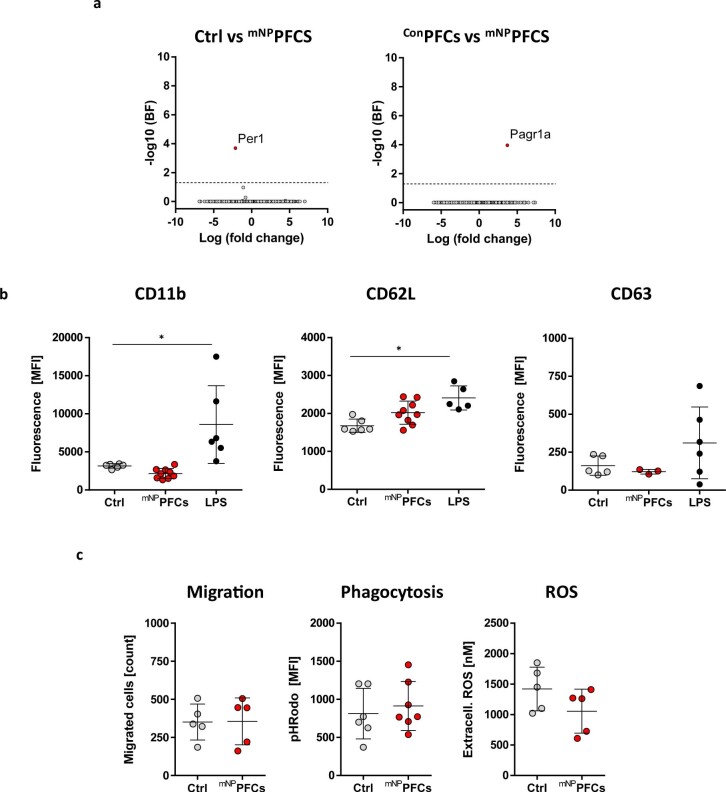
Impact of ^mNP^PFCs on murine neutrophils. **a**, Differentially expressed genes (DEGs) identified by bulk RNA sequencing of murine blood neutrophils after intravenous injection of ^mNP^PFCs, ^Con^PFCs or NaCl (Ctrl) as control. Volcano plots of DEGs for murine neutrophils of animals treated with saline compared to ^mNP^PFCs (left), or ^Con^PFCs compared to ^mNP^PFC (right). Genes marked in red are significantly upregulated with a log_2_ (fold change) greater than 1.5. BF = Bonferroni corrected p-values of the false discovery rate. In total 25655 RNA transcripts were analyzed. **b**, Cell surface expression of CD11b, CD62L, CD63 on blood derived murine neutrophils after intravenous application of NaCl (Ctrl) or ^mNP^PFCs. As a positive control, matrigel doped with 50 µg LPS was subcutaneously implanted into the neck of mice and after 24 h, neutrophils were isolated from the blood and analyzed by flow cytometry. **c**, Impact of ^mNP^PFC incubation on neutrophil migration, phagocytosis and release of reactive oxygen species (ROS). Migration was determined in vivo by assessment of the infiltration of neutrophils into a matrigel/LPS plug; phagocytosis was determined by intravenous injection of FITC-labelled *E.coli* into mice and the subsequent removal and flow cytometric analysis of blood neutrophils; extracellular ROS were measured within the cell supernatant of blood neutrophils by oxidation of DHE (dihydroethidium) followed by UPLC analysis. Data are shown as means ± SD of n = 6 (a), n = 3-9 (b) and n = 5-7 (c) independent experiments. * = p < 0.05 verified by one-way ANOVA. Source data

To investigate whether the targeting agent impacts physiological neutrophil effector functions, we first performed bulk mRNA sequencing (~45,000 genes) of human neutrophils exposed to saline (control), ^Con^PFCs or ^hNP^PFCs. However, after Bonferroni correction of the datasets, we identified just six genes that exhibited only moderately different expression levels (*BTNL3*, *CLU*, *CXCL5*, *PF4*, *PPBP* and *RGPD5*; all upregulated) when comparing the targeting PFCs to saline and only one gene when comparing to ^Con^PFCs (*RGPD5*, upregulated; Fig. 6a). As C–X–C chemokine ligand (CXCL)5 is known as a driver of neutrophil recruitment^17^, we next verified their migration toward interleukin (IL)-8 in the presence of the targeting PFCs but found no evidence for any changes in their chemotactic properties (Fig. 6b). Furthermore, neither ^hNP^PFCs nor the free peptide had any impact on human neutrophil ROS release (Fig. 6c). Additionally, we analyzed expression of the transmembrane proteins CD11b, CD63 and CD66b, reported as sensitive markers for human neutrophil activation^18–20^. Exposure of neutrophils to ^hNP^PFCs did not affect expression of these proteins, while LPS as a positive control led to significantly increased levels of CD11b and CD66b (Fig. 6d). Of note, very similar results were obtained in corresponding experiments for ^mNP^PFCs and murine neutrophils (Extended Data Fig. 9a–c). Bulk RNA sequencing of ~25,000 genes upon ^mNP^PFC injection indicated only one gene with changed expression levels compared to saline (*Per1*, downregulated) and to ^Con^PFCs (*Pagr1a*, upregulated), respectively (Extended Data Fig. 9a). Moreover, migration, ROS production and phagocytosis were also unaltered in the presence of the targeting PFCs (Extended Data Fig. 9c) as expression levels of murine activation markers CD11b, CD62L and CD63 (ref. ^21^), while again LPS significantly increased CD11b and CD63 on the cell surface of murine neutrophils (Extended Data Fig. 9b).

### Effect of inflammatory stimuli on ^NP^PFC labeling

**Fig. 7:**
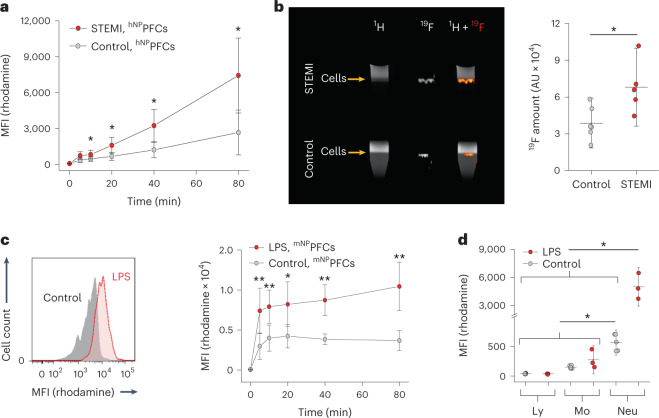
Inflammatory stimuli increase uptake of ^NP^PFCs by neutrophils. **a**, Isolated neutrophils obtained from patients with STEMI 24 h after MI (red) and from healthy controls (gray) were exposed to ^hNP^PFCs, and their uptake was determined over time by flow cytometry. **b**, Human neutrophils from patients with STEMI (top) or healthy controls (bottom) were incubated with ^hNP^PFCs, purified by density gradient centrifugation and analyzed by ^1^H/^19^F MRI. Cells are visible in ^1^H MR images as a small layer (arrow) in the bright aqueous phase on top of the black Percoll layer (left). Fluorine-19 MRI (middle) and subsequent fusion of both datasets (right) confirmed localization of the ^19^F label within the cellular layer. Quantification of ^19^F signals showed enhanced labeling of neutrophils from patients with STEMI (red) in comparison to controls (gray). **c**, Murine neutrophils isolated from mice with an inflammatory hot spot (Matrigel–LPS plug; red) or Matrigel–PBS as a control (gray) were incubated ex vivo with ^mNP^PFCs and analyzed over time by flow cytometry. Histograms (left) display representative data after 40 min, and the time course of mean fluorescence values is illustrated on the right. **d**, ^mNP^PFCs were intravenously injected in mice with a Matrigel–LPS or Matrigel–PBS (control) plug implanted 24 h before. One hour after injection, blood was withdrawn, and the in vivo uptake of ^mNP^PFCs by lymphocytes (Ly), monocytes (Mo) and neutrophils (Neu) was analyzed by flow cytometry. Data are mean ± s.d. of *n* = 5–8 (**a**), *n* = 5–6 (**b**), *n* = 4–5 (**c**) and *n* = 3–5 (**d**) independent experiments; *P < 0.05, **P < 0.01, verified two-way ANOVA (**a**,**c**,**d**) or two-sided Student’s *t*-test (**b**). Source data

**Extended Data Fig. 10:**
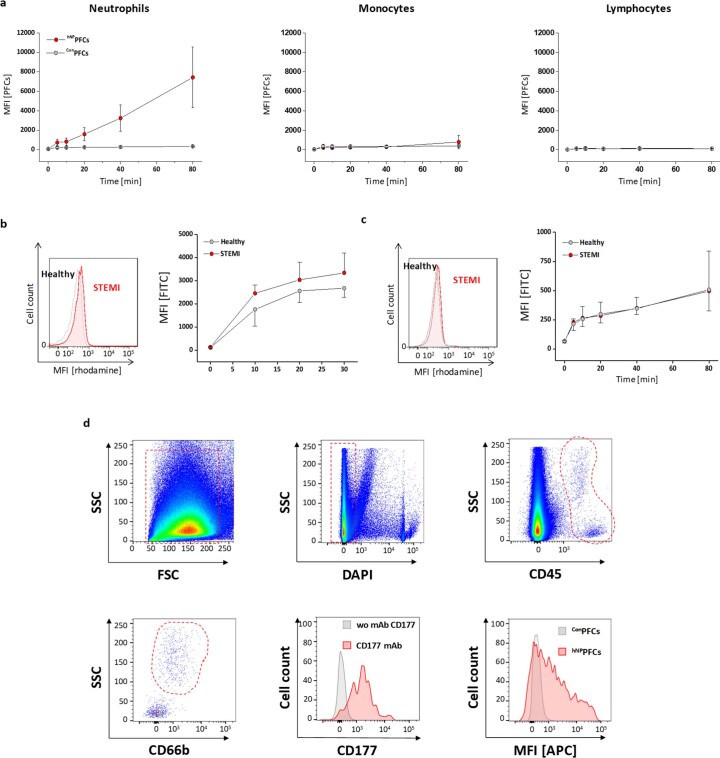
Uptake of ^hNP^PFCs by immune cells from patients. **a**, ***Uptake of*** ^***hNP***^***PFCs by immune cells from STEMI patients:*** Uptake of ^hNP^PFCs (red) or ^Con^PFCs (gray) by neutrophils, monocytes and lymphocytes from STEMI blood samples. While neutrophils show strong binding of ^hNP^PFCs in comparison to ^Con^PFCs, monocytes and lymphocytes show only negligible signals. **b** + **c**, ***Increased nanotracer uptake by STEMI patients is hNP-specific:*** **b**, Fluid-phase endocytosis by neutrophils from healthy controls as well as from STEMI patients was determined by incubation with FITC-labelled low molecular weight dextran-particles (10 kDa) over 30 min at 37 °C. At distinct time points the uptake was measured by flow cytometry indicating no significant differences between both groups. **c**, Phagocytosis was determined by incubation of neutrophils from healthy donors (gray) as well as from STEMI patients (red) with neat PFCs over 80 min at 37 °C. At distinct time points the uptake was measured via flow cytometry indicating no major differences between both groups. Left: Histogram overlays of healthy (gray) and STEMI (red) samples incubated with dextran particles (b) or PFCs (c). **d**, ***CD177 expression and*** ^***hNP***^***PFC uptake of neutrophils derived from tissue samples of explanted human hearts:*** Representative histograms of human heart samples. Dead cells were excluded from the analysis by DAPI staining. CD45 and CD11b stainings were carried out to identify neutrophils within the samples. These neutrophils exhibited both strong CD177 expression and uptake of ^hNP^PFCs. Data are means ± SD of n = 4-6 (a), n = 4-5 (b + c) or n = 3 (d) independent experiments. Source data

In a next step, we investigated whether ^NP^PFC uptake by neutrophils is altered under pathological challenges. To mirror the conditions in murine trafficking experiments after MI (Fig. 1), we used isolated neutrophils from blood of patients with STEMI (that is, ST elevation MI) obtained within the first 24 h after MI and found substantially stronger cellular uptake of ^hNP^PFCs than that of healthy volunteers (control) as demonstrated by both flow cytometry and ^19^F MRI (Fig. 7a,b). Importantly, there was no ^hNP^PFC incorporation by other blood cells from patients with STEMI (Extended Data Fig. 10a) and the elevated uptake was specific to hNP, because neither conventional PFCs nor dextran particles showed stronger internalization by neutrophils from patients with STEMI (Extended Data Fig. 10b,c).

To evaluate whether this observation is a cross-species phenomenon associated with various inflammatory stimuli, we employed a well-defined model of murine inflammation induced by subcutaneous implantation of a Matrigel plug doped with LPS^22^. Twenty-four hours after surgery, neutrophils were isolated from the blood, incubated ex vivo with ^mNP^PFCs and subsequently analyzed by flow cytometry. In line with the findings above, we observed more rapid and potent uptake of ^mNP^PFCs by murine neutrophils under LPS-stimulated conditions than under unstimulated conditions (control, Fig. 7c). Similar results were obtained in vivo: intravenous ^mNP^PFC application resulted already after 1 h in much stronger ^mNP^PFC incorporation into circulating neutrophils from LPS-challenged mice than that from healthy mice (Fig. 7d). Importantly, this effect was restricted to neutrophils, while lymphocytes and monocytes showed only minor and unaltered ^mNP^PFC uptake under inflammatory conditions.

**Fig. 8:**
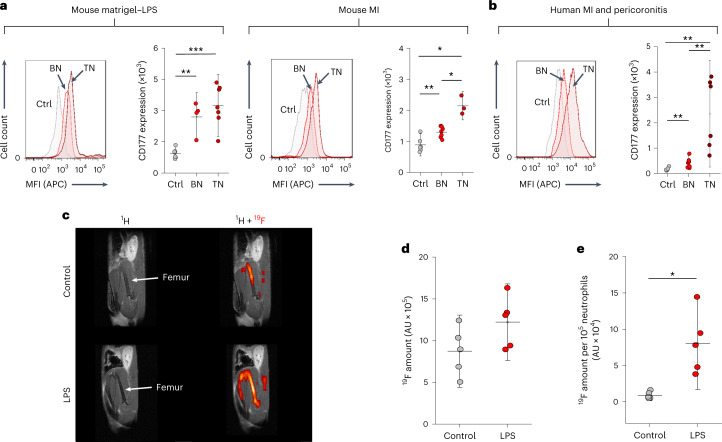
^NP^PFC loading as a readout for neutrophil-activation state. **a**, Cell surface expression of CD177 on murine neutrophils isolated from healthy controls (Ctrl, gray) after implantation of Matrigel–LPS (left) or after experimental MI (right) as determined by flow cytometry. Data were separated for circulating neutrophils isolated from the blood (BN, blood neutrophils, light red) and neutrophils obtained from the inflamed Matrigel plug or the infarcted heart (TN, tissue neutrophils, dark red). **b**, CD177 expression of human neutrophils isolated from blood of healthy controls (gray), patients with STEMI (blood neutrophils, light red) and neutrophils obtained from pericoronitis tissue specimens (tissue neutrophils). Similar to mice, CD177 expression was significantly increased in blood neutrophils compared to control values and again substantially higher in tissue neutrophils than in blood neutrophils. **c**, In vivo ^mNP^PFC labeling of bone marrow neutrophils dependent on their activation state: ^mNP^PFCs were intravenously injected into mice 24 h after implantation of Matrigel doped with LPS (bottom) or PBS as a control (top). Another 24 h later, the bone marrow was analyzed by ^1^H/^19^F MRI. **d**, Quantification of the total amount of ^19^F in the bone marrow of LPS-stimulated mice compared to the control. **e**, Normalizing the ^19^F signal to the number of neutrophils in the bone marrow demonstrated a significant increase in ^mNP^PFC uptake per cell after LPS treatment as compared to the control. The number of neutrophils in the bone marrow was determined directly after MRI by flow cytometry. Data are mean ± s.d. of *n* = 4–7 for Matrigel experiments, *n* = 3–7 for MI (**a**), *n* = 6–8 (**b**), *n* = 5 (**d**) and *n* = 5 (**e**) independent experiments; *P < 0.05, **P < 0.01, ***P < 0.001, verified by one-way ANOVA (**a**,**b**) or two-sided Student’s *t*-test (**e**). Source data

Next, we explored whether the enhanced incorporation of neutrophil-specific peptide PFCs (^NP^PFCs) under inflammatory stimulus conditions is specifically related to altered surface expression levels of their target receptor CD177. First, we analyzed neutrophils isolated from blood and excised Matrigel–LPS plugs 24 h after implantation and found that surface expression of CD177 was indeed higher in blood neutrophils from stimulated mice than in those from unstimulated mice (control, Fig. 8a, left). However, neutrophils that were obtained directly from the inflammatory focus, that is, the Matrigel–LPS plug (tissue neutrophils) tended to exhibit even higher expression levels. Similar effects were observed after experimental MI (Fig. 8a, right): again, expression levels of CD177 were elevated in neutrophils isolated from blood of mice with MI (blood neutrophils) versus those of healthy control mice but highest in neutrophils from the infarcted heart (tissue neutrophils).

For human neutrophils, we detected comparable alterations of CD177 expression in neutrophils from patients with STEMI. As shown in Fig. 8b, we observed significantly higher CD177 levels in blood neutrophils from patients with STEMI than in healthy controls. To further investigate neutrophils from human inflammatory lesions, we used tissue samples derived from surgery of the oral cavity, specifically for pericoronitis, known for substantial neutrophil infiltration^23^, and found once more the highest CD177 expression on those tissue neutrophils. Remarkably, neutrophils freshly isolated from explanted failing human hearts similarly showed strong CD177 expression and could be labeled with ^hNP^PFCs as well (Extended Data Fig. 10d), highlighting the potential of our approach to track neutrophils also in cardiac inflammatory processes in patients.

### In vivo determination of neutrophil state

In a final experimental series, we investigated whether the enhanced incorporation of ^mNP^PFCs as a consequence of CD177 upregulation can be exploited to assess the inflammatory state of neutrophils in vivo. To this end, we monitored in situ ^19^F incorporation into bone marrow neutrophils under stimulated conditions, employing again the Matrigel–LPS-based inflammation model. Twenty-four hours after plug implantation, ^mNP^PFCs were applied, and, another 24 h later, mice were subjected to ^1^H/^19^F MRI. As shown in Fig. 8c,d, we observed a substantially stronger ^19^F signal in the bone marrow upon LPS pre-activation (bottom) than in PBS-treated controls (top). This effect became even more evident when relating the detected ^19^F signal to the number of neutrophils present in the bone marrow as determined directly after MRI (Fig. 8e). This accounts for stimulated neutrophils already released from the bone marrow and revealed significantly stronger ^19^F uptake in the LPS-treated group (Fig. 8e), in line with the enhanced surface expression of CD177 upon LPS challenge (Fig. 8a). Of note, the increase in ^mNP^PFC incorporation into bone marrow neutrophils was on the same order of magnitude as the increase in circulating neutrophils observed under the same conditions (Fig. 7d), indicating that our approach is suitable to mirror the state of neutrophils in both bone marrow and blood.

## Discussion

Here, we report a new technique for global in vivo mapping of human and mouse neutrophils by equipping PFCs with peptides directed against human or murine CD177 for readout by ^19^F MRI. This approach proved to be suitable for highly specific detection of neutrophils in vitro and in vivo. We were able to label neutrophils in situ, visualize them non-invasively within their different hematopoietic niches over the entire body and track their migration to the injured heart after MI in vivo. Locoregional analysis of the data revealed the femur as the largest neutrophil reservoir as well as the main source for neutrophil release upon MI, with the diaphysis as the most active compartment. We also demonstrated that both sterile (acute MI) and nonsterile (LPS) inflammation resulted in enhanced labeling of murine and human neutrophils, which can serve as an in vivo readout for their activation state.

Neutrophils have been visualized by a variety of different imaging modalities^5,24–27^. However, optical approaches are not yet ready for clinical routine imaging, while a whole-body imaging technique such as PET provides excellent sensitivity, for example, targeted imaging with ^68^Ga-pentixafor proved useful for identification of chemokine (C–X–C motif) receptor 4 (CXCR4) expression patterns in the myocardium and systemic organs^28^, which could already be exploited for imaging-based theranostics^29^. Nevertheless, in terms of specificity, this approach is somewhat limited as CXCR4 is not only strongly expressed by neutrophils but also by monocytes^30^. Furthermore, (targeted) PET probes are usually short-lived nuclide tracers, raising difficulties in tracking neutrophils over longer periods of time, as in the present study. The widely available MRI platform provides the inherent advantage of combining excellent anatomical resolution with the opportunity for overlaying additional tissue and cell information. For visualization by MRI, to our knowledge, only neutrophils incubated ex vivo with iron oxide nanoparticles have been used^26,31^. However, the susceptibility effects induced by the re-implanted cells are rather challenging to quantify and are often difficult to differentiate from other unspecific artifacts. By contrast, our approach does not interfere with the anatomical ^1^H images, enabling precise anatomical localization of the ^19^F hot spot and easy quantification, because the ^19^F signal linearly correlates with the amount of the deposited PFCs. Decorating the PFC surface with binding peptides against human or mouse CD177 (hNP or mNP) ensured specific uptake of the targeted PFCs by neutrophils, while additional PEGylation masked the PFC droplets for passive internalization by other phagocytic immune cells^9,32,33^.

Selecting CD177 as a neutrophil-specific target had the advantage that, even though it is linked to glycosyl-phosphatidylinositol (GPI), it exhibits no transmembrane domain that can transmit signals intracellularly; therefore, binding to CD177 is unlikely to have major effects on cell activation and functionality^34^. By contrast, binding to Ly6G, a common marker for murine neutrophils, is known, for example, to modulate their migration to inflammatory foci^35^. Similarly, approaches that target receptor components of the innate immune system via *N*-formylmethionine-leucyl-phenylalanine (fMLP)^36–38^ or Fc-γ^39,40^ on the neutrophil surface are prone to alter their activation state and furthermore are not highly specific for neutrophils. Transcriptome gene expression analysis of murine and human neutrophils exposed to ^NP^PFCs, ^Con^PFCs or saline as well as additional functional analyses confirmed that our labeling approach has no critical effect on their phenotype. RNA-sequencing data of neutrophils incubated with the different compounds revealed only a few differentially expressed genes, when adjusting a threshold of 1.5-fold change in expression levels, and these alterations occurred over a very moderate range. The genes that were altered (*Per1* and *Pagr1*) in murine neutrophils are not directly linked to neutrophil function, and, among the six differentially expressed genes in human neutrophils, only *CXCL5* and *PF4* (encoding platelet factor 4) are related to neutrophil migration and function. However, we found no impact of our targeting agents on chemotaxis or ROS release of either human or murine neutrophils. In this context, it is important to note that stimulation of neutrophils with pro-inflammatory agents usually results in an order-of-magnitude higher upregulation or downregulation of several hundred genes^41–43^. In particular, genes encoding classical pro-inflammatory cytokines, chemokines and signaling pathways are upregulated such as IL-1β, tumor necrosis factor (TNF)-α, IL-6, IL-8, monocyte chemoattractant protein 1 (MCP-1) and the nuclear factor (NF)-κB pathway, but none of these were observed to be altered in RNA-sequencing data from neutrophils of both species after incubation with ^NP^PFCs. Altogether, gene expression analysis as well as functional analysis data provided no evidence that our targeting approach with ^NP^PFCs has any substantial impact on neutrophil functionality. These findings are further supported by previous observations that cross-linking of CD177 did not induce degranulation or oxidative burst^34^.

Of note, the precise functional role of CD177 in vivo still remains elusive^44^. It has been reported that its expression is upregulated in inflammatory bowel and Kawasaki diseases^45,46^. Here, we extended this finding to sterile (acute MI) and bacterial (LPS) inflammation, indicating it as a general phenomenon that might be suitable for assessing the inflammatory state of neutrophils in vivo by enhanced ^NP^PFC labeling. As CD177 is already expressed at the metamyeloid stage, our technology has the potential to provide insight into neutrophil dynamics from their formation and release from the bone marrow to migration into inflammatory foci. Although not feasible at the single-cell level, it allows us to monitor trafficking of the vast majority of neutrophils from their origin into the target tissue. Because this is also applicable to human neutrophils and PFCs have previously been evaluated in clinical trials, ^hNP^PFCs clearly offer the option for transfer into the clinical setting. Interestingly, in contrast to mice, CD177 is not present on all neutrophils in humans. In our samples, approximately 50–60% of all human neutrophils were labeled by ^hNP^PFCs. Thus, in humans, this restricts our approach to the tracking of CD177-positive neutrophils, which, on the other hand, offers the opportunity to specifically understand the biology of this large subpopulation. However, given the increased labeling under inflammatory conditions, the sensitivity of this approach would be substantially amplified under most pathophysiological conditions.

In translation to the clinical setting, our approach will allow us to not only identify hidden origins of bacterial or sterile inflammation in patients but also to unravel disease states that are on the verge of severe aggravation due to enhanced neutrophil infiltration or activation. For example, neutrophils are well known to play a pivotal role after STEMI and are recruited in the first wave after the insult into the myocardium^47^. They contribute not only to the initial tissue response but are also key players in the so-called ischemia–reperfusion injury as well as microvascular obstruction, which is known as a major reason for adverse remodeling and re-hospitalization due to heart failure in patients after STEMI^48,49^. Importantly, there are recent reports that neutrophil infiltration after MI can be beneficially modulated by metoprolol^50,51^. Thus, our approach might help to identify high-risk patients with enhanced neutrophil activation and infiltration for tailored therapy to address their specific needs, which would be of substantial clinical value. Finally, as our approach is not limited to PFCs and MRI, the hNP ligand can easily be conjugated to tracers for other imaging modalities and may be further used as a theranostic tool^29^.

## Methods

Animal experiments were performed in accordance with national guidelines on animal care and were approved by the Landesamt für Natur, Umwelt, und Verbraucherschutz (Nordrhein-Westfalen, Germany, file references 81-02.04.2017.A468 (mice), 81-02.04.2020.A290 (mice), L84-02.04.2016.A322 (pigs) and 84-02.04.2014.A232 (rats)). All studies with human samples were conducted after informed consent according to the Declaration of Helsinki and local ethics board approval (Ethikkommission, Universitätsklinikum Düsseldorf, Germany; file references 2017114486 and 2021-1635). All study participants gave written informed consent.

### Preparation of ^NP^PFCs and ^Con^PFCs

Peptides used in this study were previously identified by phage display screening approaches^11,15^. We modified the peptide sequences by adding a C-terminal cysteine for coupling reactions followed by three glycines as a spacer and an N-terminal carboxyfluorescein to enable fluorescence detection (Extended Data Fig. 1). Peptides were synthesized by Genaxxon BioScience with purity >95% (mNP, DFYKPMPNLRIT-GGG-C; related Con, SLAMFLTHSPEP-GGG-C; hNP, DLVTSKLQV-GGG-C; related Con, KQLSEMVTD-GGG-C). For TriCeps experiments, a modified hNP was used: DLVTSKLQV-GKG-C.

#### Maleimide PFCs

Nanoemulsions were composed of 20% (all wt/wt) PFCE (perfluoro-15-crown-5 ether; ABCR), 2.5% Lipoid S75 (Lipoid), 0.45% DSPE-PEG_2000_ (1,2-distearoyl-*sn*-glycero-3-phosphoethanolamine-*N*-[amino(polyethylene glycol)-2000]; Lipoid), 0.05%, maleimide-PEG_2000_-DSPE (Avanti Polar Lipids), 0.025% Lissamine-rhodamine-DHPE (Molecular Probes) and phosphate glycerol buffer up to 100%. Lipids were dissolved in chloroform and added to a round-bottom flask. Chloroform was removed in a rotary evaporator at 200 mbar and 40 °C. Thereafter, the evenly distributed lipids were resuspended in 10 mM phosphate buffer (pH 7.4), and PFCE was added dropwise. The crude emulsion was further processed on an LV1 Microfluidizer (Microfluidics) for five cycles at a process pressure of 1,000 bar.

For generation of ^NP^PFCs or ^Con^PFCs, peptides were coupled to ^Mal^PFCs. The peptides were used in fivefold molar shortfall to maleimide and linked via the free sulfhydryl group of cysteine. After incubation for 24 h at 20 °C and 750 r.p.m., the nanoemulsions were stored at 4 °C.

### Animal experiments

Animals used in this study were obtained from Janvier, housed at the central animal facility of Heinrich Heine University Düsseldorf on a 12-h light–dark cycle, fed with a standard chow diet and received tap water ad libitum. Male 10–12-week-old C57BL/6 mice (in total, *n* = 180) ranging from 20 to 30 g in body weight (BW) were used.

#### Immune cells from blood or bone marrow

Heparinized blood was withdrawn by venous puncture with a 23G cannula of the inferior vena cava. Erythrocytes were lysed by adding a fourfold volume of NH_4_Cl buffer (pH 7.4). After 10 min of incubation, samples were centrifuged at 350*g* for 10 min at 20 °C. For isolation of neutrophils from the bone marrow, mice were killed by cervical dislocation, and bones were dissected. Afterward, cells were isolated from the bone marrow using established protocols^52^.

#### Immune cells from Matrigel–LPS plugs

Mice were killed by cervical dislocation, and the Matrigel plug was carefully excised. The plug was incubated in DMEM containing 1 mg ml^−1^ collagenase II (Merck) for 10 min at 37 °C. Afterward, the sample was meshed through a cell strainer (40 µm), and isolated cells were resuspended in Miltenyi automated cell sorting (MACS) buffer.

#### Immune cells from infarcted hearts

Mice were killed, and the heart was excised and transferred into MACS buffer to flush out the blood. After mincing, samples were incubated with 1 mg ml^−1^ collagenase at 37 °C for 30 min under constant shaking and afterward meshed through a cell strainer (40 µm). To remove cardiomyocytes, samples were centrifuged at 55*g* for 5 min. The resulting supernatant containing the immune cells was used for further experiments.

#### In situ labeling of neutrophils for tracking after MI

For labeling of neutrophils before MI, mice were anesthetized (1.5% isoflurane) and 1 mmol per kg BW PFCs were injected intravenously via the tail vein on 3 consecutive days. Induction of MI was essentially carried out as previously described^53^. For inhibiting the egress of neutrophils from the bone marrow in a subset of experiments, a cocktail of neutralizing antibodies was injected i.p. 1 h before and 4 h after MI (CXCL1, CXCL2, G-CSF, GM-CSF, 50 µg each, Thermo Fisher). To generate neutropenic mice, 48 and 24 h before MI, 500 µg of the Ly6G-depletion antibody (BioXcell, clone 1A8) was injected i.p. in independent experiments.

#### Matrigel–LPS experiments

To induce defined inflammatory foci, we adopted a recently developed model of localized subcutaneous inflammation^22^. To this end, ice-cold Matrigel (Corning) was doped with LPS (1 µg µl^−1^; *Salmonella typhimurium*, Sigma-Aldrich) and subcutaneously (s.c.) implanted into the neck of the mice. Twenty-four hours after implanting the plug, neutrophils were isolated from the blood and incubated ex vivo with ^mNP^PFCs or ^Con^PFCs. In separate experiments, 24 h after implantation, ^mNP^PFCs or ^Con^PFCs were injected intravenously, and immune cells were isolated from the blood. In further experiments, 1 d after plug injection, mice received 1 mmol per kg BW ^mNP^PFCs, and, 24 h later, the bone marrow was analyzed by ^1^H/^19^F MRI. Thereafter, mice were killed, and neutrophils were isolated from the bone marrow for determination of cell numbers. In each case, immune cells were analyzed by flow cytometry.

#### ^mNP^PFC uptake by circulating immune cells and bone marrow neutrophils

Mice were kept in anesthesia (1.5% isoflurane) on a warming plate, and 1 mmol per kg BW ^mNP^PFCs or ^Con^PFCs were injected i.v. into the tail vein. One hour after injection, blood was collected from the vena cava, and immune cells were isolated. For in vivo uptake studies in the bone marrow, mice were treated as described above and killed 2 h later to isolate neutrophils from the bone marrow and analyzed by flow cytometry.

#### ^mNP^PFC uptake by immune cells isolated from the heart

^mNP^PFCs or ^Con^PFCs (3 mM per kg BW) were injected intravenously 2 h before induction of MI. One hour after MI, murine immune cells were isolated from the heart using a Langendorff digestion protocol as described previously^54^. The resulting cell fractions were analyzed by flow cytometry. Cells were stained for CD45, CD11b, Ly6C and Ly6G for 20 min at 4 °C to identify lymphoid cells (CD45^+^CD11b^−^), classical monocytes (CD45^+^CD11b^+^CD11C^+^Ly6G^−^), macrophages (CD45^+^CD11b^+^CD11C^−^Ly6G^−^) and neutrophil granulocytes (CD45^+^CD11b^+^CD11C^−^Ly6G^+^). DAPI staining was performed to exclude dead cells from analysis.

#### Immune cell isolation from pig and rat blood

To obtain immune cells from pigs, heparinized blood was withdrawn from the ear vein of animals using a 22G vein catheter as previously described^55^. For rats, heparinized blood was withdrawn by cardiac puncture of the left ventricle using a 23G cannula. For both species, erythrocytes were lysed as described above. After 10 min of incubation, samples were centrifuged at 350*g* for 10 min at 20 °C.

### Heart tissue histology and immunostaining

Neutrophils were prelabeled by i.v. injection of ^mNP^PFCs or ^Con^PFCs (3 mM per kg BW) 2 h before induction of MI, and heart samples were collected 2 or 24 h after surgery. The infarcted area was delineated by TTC staining (1%). Cryosections (40–60 µm) for MFI assessment and immunostaining were air dried and fixed with Zamboni fixative, and fluorescence images were immediately acquired. For subsequent immunostaining, tissue slices were permeabilized with Triton (1%, Sigma). Primary antibodies, including anti-Ly6G (neutrophils, 1:100), anti-MHC II (macrophages, 1:100) and anti-CD3 (T cells, 1:200), were incubated overnight at 4 °C. After three washing steps, FITC-labeled secondary antibodies were used to identify cell markers, and nuclei were counterstained with DAPI. MFI was quantified in the entire area of MI from at least six successive sections of each heart (500-µm interval). For cellular MFI, the rhodamine signal was quantified in areas where it was colocalized with the specific FITC-labeled cell markers. Micrographs were acquired with a fluorescence microscope (BX 61; Olympus) and analyzed with Fiji 1.52n^56^.

### Impact of ^mNP^PFCs on gene expression and neutrophil function

#### Gene expression

One mM per kg BW ^mNP^PFCs or saline were injected i.v. into mice, and, 2 h later, neutrophils were isolated from the bone marrow. Total RNA was isolated from purified neutrophils. For transcriptome analyses, DNase-digested total RNA samples were quantified (Qubit RNA HS Assay, Thermo Fisher), and quality control was performed by capillary electrophoresis using a fragment analyzer and the Total RNA Standard Sensitivity Assay (Agilent). All samples in this study showed high-quality RNA quality numbers (mean = 9.3). Library preparation was performed according to the manufacturer’s protocol using the Illumina Stranded mRNA Prep, Ligation kit. Briefly, 25 ng total RNA was used for mRNA capturing, fragmentation, synthesis of cDNA, adaptor ligation and library amplification. Bead-purified libraries were normalized and finally sequenced on the NextSeq 1000 system (Illumina) with a single-read setup of 1 × 100 bp. The Illumina DRAGEN FASTQ Generation tool (version 3.8.4) was used to convert the BCL files to FASTQ files as well for adaptor trimming and demultiplexing. Data analyses of FASTQ files were conducted with CLC Genomics Workbench (version 22.0.1, Qiagen). The reads of all probes were adaptor (Illumina TruSeq) and quality trimmed (using default parameters: bases below Q13 were trimmed from the end of the reads; ambiguous nucleotides, maximum 2). Mapping was done against the *Mus musculus* (mm39, GRCm39.105, 12 January 2022) and the *Homo sapiens* (hg38, GRCh38.100, 5 June 2020) genome sequences.

#### Migration

One mM per kg BW ^mNP^PFCs or saline were injected i.v., and, after 1 h, LPS-doped Matrigel was implanted s.c. into the neck of mice. After 2 h, the Matrigel plug was excised, and infiltrated neutrophils were isolated and stained with anti-CD11b and anti-Ly6G antibodies for flow cytometry.

#### Reactive oxygen species production

One mM per kg BW ^mNP^PFCs or saline were injected i.v., and, 2 h later, neutrophils were isolated from the bone marrow, and extracellular ROS was measured in the cell supernatant by oxidation of dihydroethidium followed by UPLC analysis.

#### Phagocytosis

One mM per kg BW ^mNP^PFCs or saline were injected i.v., followed by 100 µl FITC-labeled *Escherichia coli* particles (Thermo Fisher) 2 h later. Again, 2 h later, neutrophils were isolated from the blood and stained for Ly6G for 20 min at 4 °C. Thereafter, FITC labeling was determined by flow cytometry.

#### Cell surface activation markers

One mM per kg BW ^mNP^PFCs or saline were injected i.v., and, 2 h later, neutrophils were isolated from the blood and stained for CD11b, CD62L and CD63. As a positive control, mice were implanted with LPS-doped Matrigel 24 h before blood withdrawal.

#### Liver serum markers and histology of liver and spleen after ^mNP^PFC injection

^Con^PFCs, ^mNP^PFCs (each at 1 mM per kg BW) or saline were injected i.v. over 3 consecutive days. Twenty-four hours after the last injection, mice were killed, and blood samples were withdrawn to determine GLDH, AST, ALP, ALT and bilirubin levels by standard clinical procedures. Furthermore, the liver and spleen were dissected, fixed in formalin and snap frozen. Subsequently, 4-µm cryosections were cut and stained with hematoxylin and eosin as described previously^57^.

#### Biodistribution of ^mNP^PFCs

To determine the biodistribution of ^mNP^PFCs, 3 mM per kg BW ^mNP^PFCs were injected intravenously, and ^19^F signal intensities were determined in blood, liver and spleen at distinct time points after injection.

### Experiments with human blood and tissue samples

In total, blood samples from 17 patients with STEMI were used (5 female, 12 male; aged 66.9 ± 13.6 years; troponin T, 3,398 ± 3,520 ng l^−1^; creatine kinase, 933.4 ± 685.8 U l^−1^; lactate dehydrogenase, 547.8 ± 259.9 U l^−1^). Samples were analyzed 24 h after MI. Samples from the oral cavity of patients with pericoronitis (*n* = 6; 4 female, 2 male; 34.1 ± 21.7 years) and explanted human hearts (*n* = 3; 1 female, 2 male; 41.6 ± 18.2 years) were processed directly after surgery.

#### Immune cell isolation from human blood

Blood was collected from the vena brachialis, and erythrocytes were lysed as described above. After 10 min of incubation, samples were centrifuged at 350*g* for 10 min at 20 °C. For isolation of a purified neutrophil fraction, density gradient centrifugation was performed. Five milliliters of Ficoll 1.077 (Sigma-Aldrich) was layered on 5 ml Ficoll 1.119 (Sigma-Aldrich), and 20 ml of whole blood diluted 1:2 with PBS was carefully layered on the Ficoll 1.077. Samples were centrifuged at 350*g* for 20 min with low acceleration and brake. The neutrophil layer was isolated by careful aspiration and washed with PBS. Isolated cells were resuspended in MACS buffer.

#### Immune cell isolation from pericoronitis surgeries

Human pericoronitis samples were obtained through curettage of the alveolar socket, and the peridental tissue was stored in ice-cold saline. Afterward, samples were incubated in DMEM mixed with 1 mg ml^−1^ collagenase II (Merck) for 10 min at 37 °C. Samples were meshed through a cell strainer, and isolated cells were resuspended in MACS buffer.

#### Immune cell isolation from explanted human hearts

During orthotopic heart-transplant surgery in patients suffering from terminal heart failure, a tissue specimen of about ~5 g was excised from the apex of the failing heart immediately after explantation. Tissues were immediately transferred to iced BIOPS buffer as described previously^58^. For subsequent isolation of immune cells, heart samples were cut into small pieces and digested with the Multi Tissue Dissociation Kit 2 (Miltenyi; ‘adult rat heart’ protocol) by incubating for 40 min at 37 °C with the specified enzyme mix using the 37C_Multi_G program by gentleMACS. Afterward, 7.5 ml DMEM with 20% FCS was added to stop enzymatic digestion. The sample was applied to a 70-µm cell filter and washed with 3 ml DMEM, followed by centrifugation for 5 min at 300*g*. The supernatant was discarded, and cells were resuspended in 1 ml DMEM and incubated with 10 µl ^hNP^PFCs for 30 min at 37 °C on a vertical shaker. Afterward, cells were washed twice with MACS buffer and stained for CD45, CD11b and CD66b. DAPI staining was performed for exclusion of dead cells. The uptake of ^hNP^PFCs into neutrophils (CD45^+^CD11b^+^CD66b^+^) was determined by flow cytometry.

### Cell culture experiments

#### Cell lines

CHO cells (ECACC 85050302) were cultivated in DMEM high-glucose medium (Gibco, Thermo Fisher Scientific) supplemented with 10% FBS (Gibco, Thermo Fisher Scientific), 60 mg l^−1^ penicillin and 100 mg l^−1^ streptomycin (Genaxxon BioScience) at 37 °C with 5% CO_2_ in a water-saturated atmosphere.

### Magnetic resonance imaging

#### General

All experiments were performed with a vertical 9.4 T Bruker AVANCE^III^ Wide Bore NMR spectrometer (Bruker) driven by ParaVision 5.1 and operating at frequencies of 400.21 MHz for ^1^H measurements and 376.54 MHz for ^19^F measurements using a Bruker microimaging unit, Micro2.5, with actively shielded gradient sets (1.5 T m^−1^). Data were acquired using a 25-mm quadrature ^19^F resonator with one channel tunable to both ^1^H and ^19^F. Mice were anesthetized with 1.5% isoflurane and kept at 37 °C. After acquisition of morphological ^1^H images, the resonator was tuned to ^19^F, and anatomically matching ^19^F images were recorded essentially as described previously^59^.

For whole-body images, mice were repositioned for coverage of thorax and brain, and abdomen and hindlimbs, respectively. Slice packages were placed in the axial direction, and datasets were subsequently merged using the 3D visualization software Amira (Mercury Computer Systems). Scan details are as follows: ^1^H rapid acquisition with relaxation enhancement (RARE), repetition time (TR) = 3,500 ms, field of view (FOV) = 2.56 × 2.56 cm^2^, matrix = 256 × 256, slice thickness (ST) = 1 mm, acquisition time (*t*_Acq_) = 1.24 min; ^19^F 3D RARE, TR = 2,500 ms, FOV = 2.56 × 2.56 cm^2^, matrix = 64 × 64, ST = 45 mm, *t*_Acq_ = 1 h.

#### Bone marrow

Slice packages were placed in sagittal orientation to cover the complete bone marrow in the tibia and femur in both legs using the following scan details: ^1^H RARE, TR = 2,000 ms, FOV = 4.00 × 2.56 cm^2^, matrix = 256 × 256, ST = 1 mm, *t*_Acq_ = 1 min; ^19^F RARE, TR = 2,500 ms, FOV = 4.00 × 2.56 cm^2^, matrix = 64 × 64, ST = 3 mm, *t*_Acq_ = 10 min.

#### Cardiac

Images of mouse hearts were acquired in short-axis orientation using a retrospectively triggered fast low-angle shot cine sequence (IntragateFLASH, Bruker) as described previously^60^. Thereafter, hearts were excised, washed and fixed with PFA for 3D high-resolution post-mortem MRI: ^1^H FISP, TR = 4 ms, FOV = 1.00 × 1.00 × 1.00 cm³, matrix = 128 × 64 × 128, *t*_Acq_ 151 min; ^19^F RARE, TR = 2,500 ms, FOV = 1.00 × 1.00 × 1.00 cm³, matrix = 32 × 32 × 32, *t*_Acq_ = 10 h.

Analysis of biodistribution was carried out as described previously^61^.

#### Isolated cells

After the incubation period, cells were subjected to density gradient centrifugation to separate PFC-loaded cells from free PFCs. Afterward, samples were analyzed by MRI to determine the ^19^F signal within the cell layer as described previously^61^.

#### Perfluorocarbons

For evaluation of ^19^F content, 10 µl of the nanoemulsion was transferred into PCR tubes and measured with the following parameters: ^1^H RARE, TR = 3,500 ms, RARE factor 16, FOV = 2.56 × 2.56 cm^2^, matrix = 128 × 128, ST = 1 mm, *t*_Acq_ = 1 min; ^19^F RARE, TR = 2,500 ms, RARE factor 32, FOV = 2.56 × 2.56 cm^2^, matrix = 32 × 32, ST = 1 mm, *t*_Acq_ = 5 min.

#### Data analysis

MRI data were analyzed as described previously^62^^,^^63^.

### Flow cytometry

#### General

Flow cytometry was performed with a FACSCanto II (BD Biosciences) or LSRFortessa (BD Biosciences). Cells were gated with appropriate FSC–SSC settings and thresholds for excluding debris. To omit dead cells, samples were stained with 1 µg ml^−1^ DAPI (Merck). For analysis, cells were gated with FACSDiva 6 or FlowJo 7.1, and MFI and/or the number of positive cells was determined, depending on the experiment.

Human immune cells were discriminated by staining for CD45 (BioLegend, clone HI30), CD11b (BD Biosciences, clone ICRF44), CD14 (BioLegend, clone M5E2) and CD16 (BD Biosciences, clone 3G8) (lymphocytes, CD45^+^CD11b^−^CD16^−^; classical monocytes, CD45^+^CD11b^+^CD14^++^CD16^−^; non-classical monocytes, CD45^+^CD11b^+^CD14^+^CD16^++^; neutrophils, CD45^+^CD11b^+^CD16^+^). Murine immune cells were discriminated by staining for CD45 (BD Biosciences, clone 30-F11), CD11b (BioLegend, clone M1/70), Ly6G (BD Biosciences, clone 1A8), Ly6C (BioLegend, clone HK1.4) and F4/80 (BioLegend, clone BM8) (lymphocytes, CD45^+^CD11b^−^Ly6G^−^; classical monocytes, CD45^+^CD11b^+^Ly6G^−^Ly6C^hi^; non-classical monocytes, CD45^+^CD11b^+^Ly6G^−^Ly6C^lo^; neutrophils, CD45^+^CD11b^+^Ly6G^+^; eosinophils, CD45^+^CD11b^+^Ly6G^+^SSC^hi^FSC^lo^). Both human and murine cells were stained for 20 min at 4 °C, followed by washing with 200 µl MACS buffer.

If not mentioned otherwise, neutrophil-specific peptide or Con (both at 1 µg ml^−1^) were incubated for 20 min at 4 °C, while ^NP^PFCs or ^Con^PFCs were incubated at a concentration of 10 µl ml^−1^ for the indicated period of time, followed by two washing steps with 200 µl MACS buffer.

Immune cells from rat and pig were discriminated with appropriate forward and side scattering and rat immune cells additionally by CD11b (BD Biosciences, clone WT.5) staining.

#### Cell lines

CHO cells were gated with appropriate FSC–SSC settings. Approximately 1 × 10^5^ cells were stained with anti-CD177 monoclonal antibody (BD Biosciences, clone Y127) and 1 µg ml^−1^ hNP or Con for 20 min at 4 °C. Afterward, samples were washed twice with MACS buffer and analyzed for CD177, hNP and Con binding.

### Experiments with cells

#### Binding of hNP or mNP to immune cells

Cells were isolated from blood, resuspended in 100 µl and transferred to a 96-well plate, resulting in 1 × 10^5^ cells in each well. Subsequently, cells were incubated with or without peptides. Their binding was analyzed by flow cytometry via detection of their fluorescence label.

#### Binding of free mNP to murine neutrophils

A total of 1 × 10^5^ bone marrow neutrophils were incubated with increasing amounts of mNP. To investigate putative conjugation effects, we coupled mNP to eight-arm PEG_2000_-maleimide (Sigma-Aldrich). mNP (or Con) was used at a twofold molar excess to maleimide for loading all binding sites with mNP or Con. Coupling was carried out at room temperature for 24 h with constant shaking. Afterward, 1 × 10^5^ bone marrow neutrophils were incubated with 1 µg ml^−1^ of the constructs, and uptake was determined by flow cytometry.

For identification of the surface receptor for hNP on neutrophil granulocytes, coupling of the peptide to TriCeps and cell incubation were carried out according to manufacturer’s instructions (Dualsystems)^16^. Samples were subsequently analyzed by Dualsystems.

For transient transfection of CHO cells, 2.5 × 10^5^ cells were seeded in six-well plates. Twenty-four hours later, the medium was refreshed. One µg plasmid DNA^64^ (human CD177) and 4 µl PEI MAX (Polysciences) were suspended in 100 µl saline, incubated for 15 min at room temperature and subsequently added to the wells. The medium was replaced after 24 h, and cells were cultivated further for 24 or 48 h. Thereafter, cells were detached with PBS with 2.5 mM EDTA, washed and resuspended in MACS buffer. Approximately 1 × 10^5^ cells were stained with monoclonal antibodies against CD177 or hNP or Con and analyzed by flow cytometry.

#### Cell surface expression of CD177

Neutrophils were isolated from the blood of healthy mice as well as 24 h after Matrigel implantation or induction of MI and also directly from the inflammatory hot spot (Matrigel or infarct area). For human studies, neutrophils were isolated from the blood of healthy individuals and patients 24 h after STEMI as well as from tissue samples from oral surgeries (pericoronitis). Isolated immune cells were transferred into 96-well plates, stained and analyzed by flow cytometry.

#### Internalization of hNP into neutrophils

The pH-sensitive pHrodo maleimide dye (Thermo Fisher) was mixed with hNP at an equal molar ratio in PBS and incubated at room temperature for 1 h at 700 r.p.m. to enable the conjugation of hNP and pHrodo. Afterward, isolated neutrophils were incubated with 1 µg ml^−1^ of the pHrodo–hNP construct at 4 °C or 37 °C for 30 min. At distinct time points, cell samples were washed twice with MACS buffer, and uptake of the pHrodo–hNP construct was determined via its fluorescence signal by flow cytometry.

#### Binding and internalization of ^NP^PFCs

A total of 1 × 10^6^ cells were incubated with 10 µl of the emulsion over a period of 80 min at 37 °C. At distinct time points, 50 µl of the samples were transferred into 2 ml of ice-cold MACS buffer and analyzed for rhodamine fluorescence by flow cytometry.

To determine PFC uptake by human neutrophils by ^19^F MRI, cells were isolated from 10 ml of whole blood by density centrifugation. Subsequently, neutrophils (5 × 10^6^) were resuspended in 3 ml DMEM and incubated for up to 8 h at 37 °C under constant motion with 10 µl ^hNP^PFCs. Afterward, cells were centrifuged at 350*g*, washed three times with PBS, resuspended in 1 ml MACS buffer, purified by Percoll gradient centrifugation and analyzed by ^1^H/^19^F MRI.

##### Fluorine-19 MRI of murine neutrophils

A total of 4 × 10^6^ neutrophils isolated from bone marrow with the EasySep Mouse Neutrophil Enrichment Kit (Stemcell Technologies) were resuspended in 1 ml DMEM and incubated for 3 h with 50 µl ^mNP^PFCs or ^Con^PFCs at 37 °C. Afterward, cells were washed three times with PBS, fixed with PFA, pelleted by centrifugation and analyzed by ^1^H/^19^F MRI.

##### Fluorescence microscopy of human neutrophils

Neutrophils (1 × 10^6^) were incubated with 50 µl ml^−1^ ^hNP^PFCs or ^Con^PFCs for 60 min at 37 °C, washed three times with MACS buffer, centrifuged onto a glass plate, fixed with 0.5% PFA and stained with 1 µg ml^−1^ DAPI to visualize nuclei. Finally, cells were embedded in MOWIOL and studied by confocal microscopy (Zeiss LSM 710 Meta, Zeiss). Images were analyzed using Fiji 1.52n^56^.

#### hNP competition experiment

A total of 1 × 10^6^ human neutrophils were incubated in 1 ml DMEM at 4 °C with or without 5 µg ml^−1^ hNP for 30 min. Subsequently, 10 µl ^hNP^PFCs were added, and, at defined time points, 50 µl of the cell suspension was transferred into 2 ml ice-cold FACS buffer. Cells were analyzed by flow cytometry.

#### Analysis of reactive oxygen species

A total of 5 × 10^6^ human neutrophils were incubated in 10 ml DMEM with or without 1 µg ml^−1^ peptide or 50 µl ml^−1^ emulsion. At distinct time points, cells were pelleted, and 1 ml of the supernatant was immediately frozen at −80 °C. Subsequently, the amount of ROS was determined by chemiluminescence analysis. Murine neutrophils were isolated from ^mNP^PFC-treated or NaCl-treated animals, and 1 × 10^5^ cells were incubated with dihydroethidium–HBSS buffer (20 µM) for 30 min at 37 °C. After centrifugation, 80 µl supernatant was used to determine ROS by UPLC measurement (Waters Acquity Bio H-Class with 2475 FLD Detector). To this end, gradients A and B (0.1% trifluoroacetic acid in 1 l water and acetonitrile, respectively) at a flow rate of 0.26 ml min^−1^ at 17 °C were used. Dihydroethidium was excited and detected at 480 nm and 580 nm, respectively.

#### Migration of neutrophils

A total of 1 × 10^6^ isolated human neutrophils were incubated with or without 50 µl ^hNP^PFCs for 1 h at 37 °C in 1 ml DMEM. After washing with DMEM, 1 × 10^5^ of these cells were placed in a Boyden chamber containing 200 µl DMEM. The lower part of the chamber contained 100 ng ml^−1^ IL-8 in 1 ml DMEM. After 1 h in an incubator, the flow through was collected, and the number of neutrophils was counted by flow cytometry. Murine neutrophils were isolated from Matrigel–LPS plugs and stained for Ly6G. Afterward, cells were washed twice, and the number of neutrophils was determined by flow cytometry.

#### Analysis of cell surface activation markers

A total of 1 × 10^6^ isolated human neutrophils were incubated with 50 µl ml^−1^ ^hNP^PFCs, 1 µg ml^−1^ LPS or only DMEM for 1 h at 37 °C followed by intense washing with MACS buffer. Afterward, 1 × 10^5^ cells were transferred into 96-well plates and stained for CD11b and CD63 (eBioscience, clone HSC6) or CD66b (eBioscience, clone G10F5) for 20 min at 4 °C. After two washing steps with MACS buffer, cells were analyzed by flow cytometry. A total of 1 × 10^5^ isolated murine neutrophils were transferred into a 96-well plate and stained for CD11b, CD62L and CD63 for 20 min at 4 °C. After two washing steps with MACS buffer, cells were analyzed by flow cytometry.

#### Endocytic properties of neutrophils

Isolated human neutrophils were incubated for 30 min with 1 µg ml^−1^ 10 kDa FITC-labeled dextran particles (fluid-phase endocytosis) or for 80 min with 10 µl ml^−1^ PFCs. At distinct time points, samples were taken and transferred into 2 ml ice-cold PBS. Afterward, cells were centrifuged at 300*g* for 10 min, resuspended in MACS buffer and analyzed by flow cytometry. Murine neutrophils were isolated from ^mNP^PFC-treated or NaCl-treated animals and stained for Ly6G for 20 min at 4 °C. After two washing steps, phagocytosis of FITC-conjugated *E. coli* particles was determined by flow cytometry.

#### Isolation of total RNA

Human blood samples were treated ex vivo with ^hNP^PFCs or NaCl for 1 h, and afterward neutrophils and monocytes were isolated with the blood neutrophil-isolation kit (Miltenyi) or the monocyte-isolation kit (Stemcell) according to the manufacturer’s protocol. Murine neutrophils and monocytes were isolated from ^mNP^PFC- or NaCl-treated animals from the bone marrow with isolation kits (both from Stemcell) according to the manufacturer’s protocol. Cell disruption was carried out with 100 µl RLT Plus buffer (Qiagen), and afterward RNA was isolated according to the manufacturer’s protocol (Zymo Research, RNA Clean & Concentrator).

#### SPR analysis of the human and murine neutrophil-specific peptides

The interaction between human and murine neutrophils and monocytes with NPs was analyzed using a Biacore X100 system equipped with a CM5 sensor chip (Cytiva). Immobilization of NPs to the chip surface was performed by activation of carboxymethyl groups of the CM5 chip and introduction of reactive disulfide groups by reaction with EDC–NHS and PDEA (Cytiva), followed by covalent binding of the NPs via the C-terminal free sulfhydryl group (260 µg, 5 µl min^−1^) and blockage of excess carboxyl groups. Afterward, human and murine neutrophils and monocytes (analytes) were flowed over the immobilized ligand surface to record the binding response of the analytes to the ligand. After 60 s of analyte association, the chip surface was regenerated for a period of 300 s by dissociation of the analytes with running buffer. Additionally, increasing numbers of neutrophils were flushed over the immobilized peptide with a flow rate of 30 µl min^−1^, and dissociation was measured for an identical period. The final report point, expressed in relative response units of the stability point, was calculated by subtracting the reference from the ligand response unit, indicating the level of interaction and comparative binding affinity. HBS-EP (10 mM HEPES, pH 7.4, 150 mM NaCl, 3.4 mM EDTA, 0.005% P20) and DPBS plus 0.005% P20 (Sigma-Aldrich) buffers at 25 °C were used in all experiments as binding and running buffers, respectively.

### Characterization of ^NP^PFCs and ^Con^PFCs

#### Fluorescence

For fluorescence measurements, 10 µl PFCs were spotted on a glass plate and analyzed with an IVIS Lumina II system (PerkinElmer; GFP excitation and emission filters, 0.5-s excitation).

Dynamic light scattering and the *ζ* potential were measured as described previously^9,32^ at 25 °C using a Nanotrac Wave II analyzer (Microtrac) driven by Microtrac FLEX Software 3.4.

#### Cryo-transmission electron microscopy

PFCs were diluted with sample buffer to minimize particle aggregation and therefore enable proper size measurements, which were carried out as previously described^12^.

### Statistics

No statistical methods were used to predetermine sample size. Experiments were not randomized, and the investigators were not blinded during experiments and outcome assessment. Unless otherwise indicated, all values are given as mean ± s.d. Statistical analysis was performed using OriginPro 2016 (OriginLab). Data were tested for Gaussian distribution using the D’Agostino and Pearson omnibus normality test. For comparison of parameters between the groups, a Student’s *t*-test or one- or two-way ANOVA was used.

#### RNA-sequencing data

FASTQ files were analyzed with CLC Genomics Workbench (version 22.0.1, Qiagen). The reads of all probes were adaptor (Illumina TruSeq) and quality trimmed (using the default parameters: bases below Q13 were trimmed from the end of the reads; ambiguous nucleotides, maximum 2). Mapping was done against *M. musculus* (mm39, GRCm39.105, 12 January 2022) and *H. sapiens* (hg38, GRCh38.100, 5 June 2020) genome sequences, respectively. After grouping samples (for *n* = 3 biological replicates each) according to the individual experimental conditions, statistical differential expression was determined using the Differential Expression for RNA-Seq tool (version 2.6). The resulting *P* values were corrected for multiple testing by FDR and Bonferroni correction. *P* values ≤0.05 were considered significant.

### Reporting summary

Further information on research design is available in the Nature Portfolio Reporting Summary linked to this article.

### Supplementary information

Supplementary Information Supplementary Table 1 and Figs. 1 and 2.

Reporting Summary

### Source data

Source Data Fig. 1 Statistical source data.

Source Data Fig. 2 Statistical source data.

Source Data Fig. 3 Statistical source data.

Source Data Fig. 4 Statistical source data.

Source Data Fig. 5 Statistical source data.

Source Data Fig. 6 Statistical source data.

Source Data Fig. 7 Statistical source data.

Source Data Fig. 8 Statistical source data.

Source Data Extended Data Fig. 2 Statistical source data.

Source Data Extended Data Fig. 3 Statistical source data.

Source Data Extended Data Fig. 5 Statistical source data.

Source Data Extended Data Fig. 6 Statistical source data.

Source Data Extended Data Fig. 8 Statistical source data.

Source Data Extended Data Fig. 9 Statistical source data.

Source Data Extended Data Fig. 10 Statistical source data.

## Data Availability

All data supporting the findings of this study are available within the article and its Supplementary Information. RNA-sequencing data have been uploaded to the Gene Expression Omnibus (accession no. GSE217910). Raw MRI data are available from the corresponding author. Source data are provided with this paper.
