# Supplementary material for: Non-invasive mapping of systemic neutrophil dynamics upon cardiovascular injury
Source: Nat Cardiovasc Res. 2023 Feb 6;2(2):126–43. doi: 10.1038/s44161-022-00210-w (PMC11357992; doi:10.1038/s44161-022-00210-w)

nature cardiovascular research

Article

https://doi.org/10.1038/s44161-022-00210-w

Non-invasive mapping of systemic neutrophil dynamics upon cardiovascular injury

In the format provided by the authors and unedited

Supplementary Table 1

Full list of enriched proteins for hNP compared to transferrin identified in TriCeps experiment

Protein	Description	Log2(FC)	adj. P value
BASI	Basigin	1.89	$1.69*10^{-3}$
BST1	ADP-ribosyl cyclasecyclic ADP-ribose hydrolase 2	2.23	$8.97*10^{-5}$
CD177	CD177 antigen	5.72	$8.55*10^{-6}$
CD44	CD44 antigen	2.44	$4.23*10^{-5}$
CD59	CD59 glycoprotein	3.12	$4.23*10^{-5}$
CEAM6	Carcinoembryonic antigen-related cell adhesion molecule 6	2.59	$2.34*10^{-4}$
CEAM8	Carcinoembryonic antigen-related cell adhesion molecule 8	2.83	$1.13*10^{-4}$
CLC5A	C-type lectin domain family5 member A	4.04	$4.23*10^{-5}$
CTL2	Choline transporter-like protein 2	2.00	$5.04*10^{-3}$
CXCR1	C-X-C chemokine receptor type 1	2.76	$4.70*10^{-4}$
DAF	Complement decay-accelerating factor	1.95	$3.13*10^{-3}$
EMP3	Epithelial membrane protein 3	3.77	$2.73*10^{-5}$
FCG3A	Low affinity immunoglobulin gamma Fc region receptor III-A	2.08	$2.06*10^{-4}$
FCG3B	Low affinity immunoglobulin gamma Fc region receptor III-B	2.85	$4.56*10^{-4}$
ICAM3	Intercellular adhesion molecule 3	3.63	$2.92*10^{-5}$
LEUK	Leukosialin	3.72	$3.04*10^{-5}$
LYAM1	L-selectin	3.57	$4.06*10^{-5}$
MCP	Membrane cofactor protein	1.93	$2.88*10^{-4}$
MEGF9	Multiple epidermal growth factor-like domains protein 9	2.08	$2.35*10^{-4}$
MAG	Maltase-glucoamylase, intestinal	2.56	$4.06*10^{-5}$
PECA1	Platelet endothelial cell adhesion molecule	3.45	$6.23*10^{-4}$
PTPRC	Receptor-type tyrosine-protein phosphatase C	2.46	$1.17*10^{-4}$
PTPRJ	Receptor-type tyrosine-protein phosphatase eta	2.98	$5.94*10^{-5}$
UPAR	Urokinase plasminogen activator surface receptor	1.95	$3.46*10^{-4}$

p values were verified by one-way ANOVA.

Supplementary Figure 1: Murine immune cell gating strategy

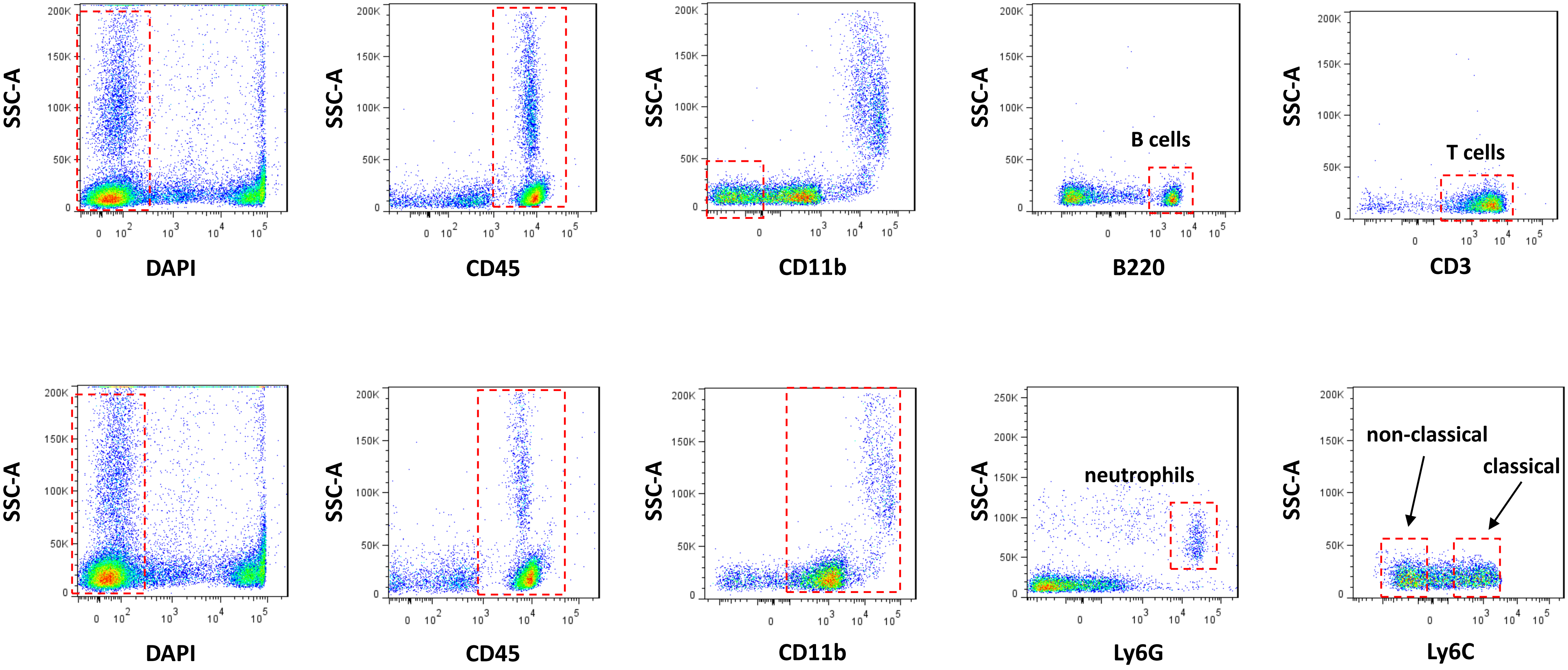

Supplementary Figure 2: Human immune cell gating strategy

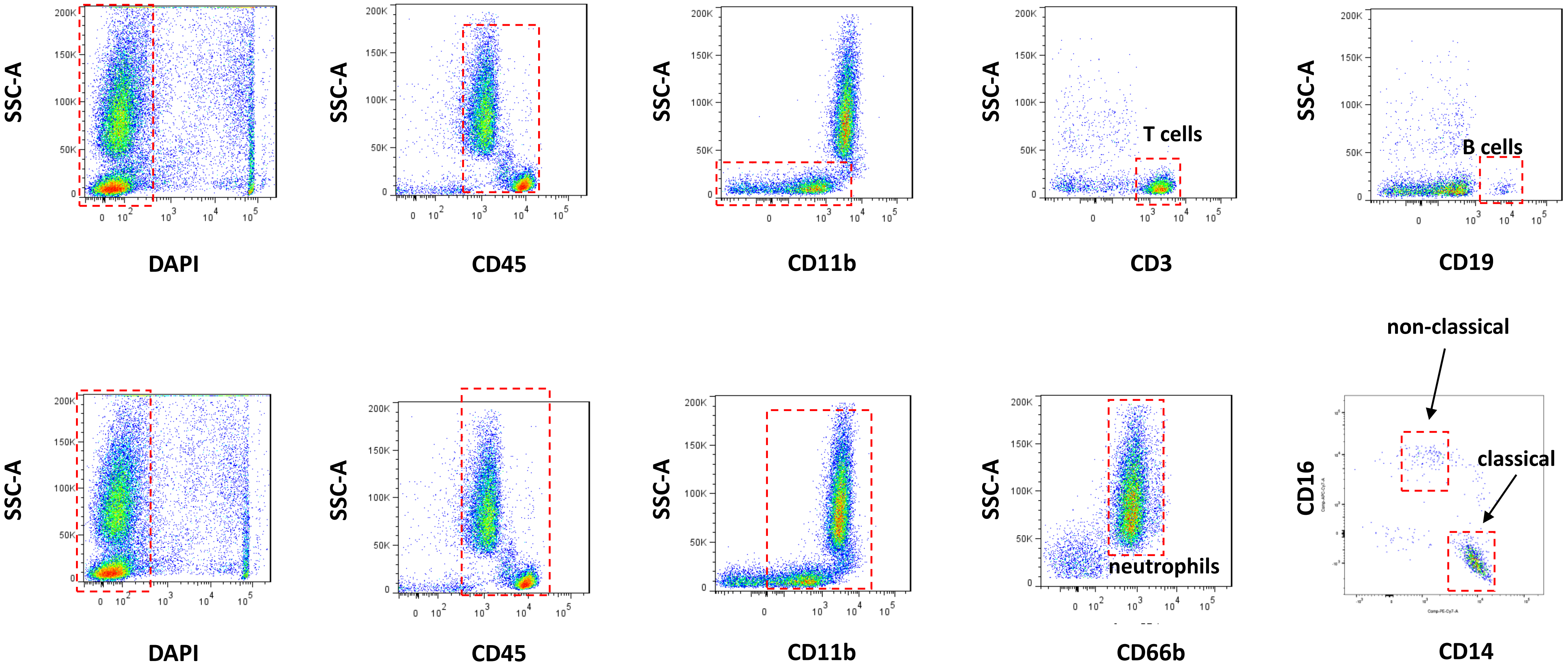

Supplement: Supplementary file 1 — Supplementary Table 1 and Figs. 1 and 2. [file 44161_2022_210_MOESM1_ESM.pdf]
