# Supplementary material for: Non-invasive mapping of systemic neutrophil dynamics upon cardiovascular injury
Source: Nat Cardiovasc Res. 2023 Feb 6;2(2):126–43. doi: 10.1038/s44161-022-00210-w (PMC11357992; doi:10.1038/s44161-022-00210-w)
Supplement: Supplementary file 2 — Reporting Summary [file 44161_2022_210_MOESM2_ESM.pdf]

nature portfolio

Corresponding author(s): Prof. Dr. Ulrich Flögel

Last updated by author(s): Dec 21, 2022

Reporting Summary

Nature Portfolio wishes to improve the reproducibility of the work that we publish. This form provides structure for consistency and transparency in reporting. For further information on Nature Portfolio policies, see our Editorial Policies and the Editorial Policy Checklist.

Statistics

For all statistical analyses, confirm that the following items are present in the figure legend, table legend, main text, or Methods section.

n/a	Confirmed	
☐	☒	The exact sample size (n) for each experimental group/condition, given as a discrete number and unit of measurement
☐	☒	A statement on whether measurements were taken from distinct samples or whether the same sample was measured repeatedly
☐	☒	The statistical test(s) used AND whether they are one- or two-sided<br>*Only common tests should be described solely by name; describe more complex techniques in the Methods section.*
☒	☐	A description of all covariates tested
☐	☒	A description of any assumptions or corrections, such as tests of normality and adjustment for multiple comparisons
☐	☒	A full description of the statistical parameters including central tendency (e.g. means) or other basic estimates (e.g. regression coefficient) AND variation (e.g. standard deviation) or associated estimates of uncertainty (e.g. confidence intervals)
☐	☒	For null hypothesis testing, the test statistic (e.g. F, t, r) with confidence intervals, effect sizes, degrees of freedom and P value noted<br>*Give P values as exact values whenever suitable.*
☒	☐	For Bayesian analysis, information on the choice of priors and Markov chain Monte Carlo settings
☒	☐	For hierarchical and complex designs, identification of the appropriate level for tests and full reporting of outcomes
☐	☒	Estimates of effect sizes (e.g. Cohen's d, Pearson's r), indicating how they were calculated

Our web collection on statistics for biologists contains articles on many of the points above.

Software and code

Policy information about availability of computer code

Data collection	ParaVision 5.1, FACS Diva 6, Microtrac Flex Software 3.4,
Data analysis	ParaVision 5.1, OriginPro 2016, Amira 4.0, FlowJo 7.1, DRAGEN FASTQ Generation tool 3.8.4, CLC Genomics Workbench 22.0.1, Fiji 1.52n

For manuscripts utilizing custom algorithms or software that are central to the research but not yet described in published literature, software must be made available to editors and reviewers. We strongly encourage code deposition in a community repository (e.g. GitHub). See the Nature Portfolio guidelines for submitting code & software for further information.

Data

Policy information about availability of data

All manuscripts must include a data availability statement. This statement should provide the following information, where applicable:

- Accession codes, unique identifiers, or web links for publicly available datasets
- A description of any restrictions on data availability
- For clinical datasets or third party data, please ensure that the statement adheres to our policy

All data necessary to understand and assess the conclusions of the manuscript are present in the paper or the supplementary materials. RNA-seq data have been uploaded to the Gene Expression Omnibus (accession no. GSE217910). Raw imaging data are available from the corresponding authors. Source data are provided with this paper.

Field-specific reporting

Please select the one below that is the best fit for your research. If you are not sure, read the appropriate sections before making your selection.

☒ Life sciences ☐ Behavioural & social sciences ☐ Ecological, evolutionary & environmental sciences

For a reference copy of the document with all sections, see nature.com/documents/nr-reporting-summary-flat.pdf

Life sciences study design

All studies must disclose on these points even when the disclosure is negative.

Sample size	No statistical methods were used to predetermine sample size. Sample size was approximated and derived from extensive publications within the cardiovascular field which were based on previous a priori power testing.
Data exclusions	No data were excluded.
Replication	All attempts at replication were successful. Experiments without quantification were repeated at least three times.
Randomization	Animals were randomly assigned to experimental groups (= allocation was random within the required age margin).
Blinding	All RNA-seq were performed blinded, as they were performed in an unbiased fashion. Histological stainings were also blinded. In most cases, blinding was not feasible because the studied phenotypes allowed unambiguous identification of the treatment. Here, OriginPro was used for semi-automatic unbiased quantification of data based on experiments performed by the same investigator.

Reporting for specific materials, systems and methods

We require information from authors about some types of materials, experimental systems and methods used in many studies. Here, indicate whether each material, system or method listed is relevant to your study. If you are not sure if a list item applies to your research, read the appropriate section before selecting a response.

Materials & experimental systems

n/a	Involved in the study	
☐	☒	Antibodies
☐	☒	Eukaryotic cell lines
☒	☐	Palaeontology and archaeology
☐	☒	Animals and other organisms
☐	☒	Human research participants
☒	☐	Clinical data
☒	☐	Dual use research of concern

Methods

n/a	Involved in the study	
☒	☐	ChIP-seq
☐	☒	Flow cytometry
☒	☐	MRI-based neuroimaging

Antibodies

Antibodies used

If not else mentioned in the method part, all antibodies are used in a 1:100 dilution

Human:
CD45, Biolegend, clone HL30, Cat 304002
CD11b, BD Biosciences, clone ICRF44, Cat 561015
CD16, BD Biosciences, clone 3G8, Cat 560918
CD14, Biolegend, clone M5E2, Cat 301807

Mouse
CD45, BD Biosciences, clone 30-F11, Cat 557235
CD11b, Biolegend, clone M1/70, Cat 101209
Ly6G, BD Biosciences, clone 1A8, Cat 560601
G-CSF, Thermo Fisher, clone 67604, Cat MA5-23758
GM-CSF, Thermo Fisher, clone MP122E9, Cat MA5-23799
CXCL-2, Thermo Fisher, clone 40605, Cat MA5-23737
CXCL-1, Thermo Fisher, clone 48415, Cat MA5-23745
CD177, BD Biosciences, clone Y127, Cat 566599
Ly6G, BioXcell, clone 1A8, Cat BE0075-1
Ly6C, Biolegend, clone HK1.4, Cat 128015
F4/80, Biolegend, clone BM8, Cat 123115
CD62L, BD Biosciences, clone MEL-14, Cat 15831469
CD63 BD Biosciences, clone NVG-2, Cat 564222

Rat
CD11b, BD Biosciences, clone WT.5, Cat 562102

Validation

Human:
CD45 --> relevant citations:
1. Thomas M. 1989. Annu. Rev. Immunol. 7:339
2. Trowbridge I, et al. 1994. Annu. Rev. Immunol.12:85

CD11b -->relevant citations:
1. Barclay NA, Brown MH, Birkeland ML, et al, ed. The Leukocyte Antigen FactsBook. San Diego, CA: Academic Press; 1997
2. David A, Kacher Y, Specks U, Aviram I. Interaction of proteinase 3 with CD11b/CD18 (beta2 integrin) on the cell membrane of human neutrophils. J Leukoc Biol. 2003; 74(4):551-557
3. Hogg N, Horton MA. Myeloid antigens: New and previously defined clusters. In: McMichael AJ. A.J. McMichael .. et al., ed. Leucocyte typing III : white cell differentiation antigens. Oxford New York: Oxford University Press; 1987; :576-602
4. Hogg N, Palmer DG, Revell PA. Mononuclear phagocytes of normal and rheumatoid synovial membrane identified by monoclonal antibodies. Immunology. 1985; 56(4):673-681
5. Knapp W. W. Knapp .. et al., ed. Leucocyte typing IV : white cell differentiation antigens. Oxford New York: Oxford University Press; 1989; :1-1182
6. Zola H. Leukocyte and stromal cell molecules : the CD markers. Hoboken, N.J.: Wiley-Liss; 2007

CD16 -->relevant citations:
1. Barclay NA, Brown MH, Birkeland ML, et al, ed. The Leukocyte Antigen FactsBook. San Diego, CA: Academic Press; 1997
2. Fleit HB, Wright SD, Unkeless JC. Human neutrophil Fc gamma receptor distribution and structure. Proc Natl Acad Sci U S A. 1982; 79(10):3275-3279
3. Knapp W. W. Knapp .. et al., ed. Leucocyte typing IV : white cell differentiation antigens. Oxford New York: Oxford University Press; 1989; :1-1182
4. Roederer M, Kantor AB, Parks DR, Herzenberg LA. Cy7PE and Cy7APC: bright new probes for immunofluorescence. Cytometry. 1996; 24(3):191-197
5. Stroncek DF, Skubitz KM, Plachta LB, et al. Alloimmune neonatal neutropenia due to an antibody to the neutrophil Fc-gamma receptor III with maternal deficiency of CD16 antigen. Blood. 1991; 77(7):1572-1580
6. van Vugt MJ, van den Herik-Oudijk IE, van de Winkle JG. Binding of PE-CY5 conjugates to the human high-affinity receptor for IgG (CD64). Blood. 1996; 88(6):2358-2361
7. Wirthmueller U, Kurosaki T, Murakami MS, Ravetch JV. Signal transduction by Fc gamma RIII (CD16) is mediated through the gamma chain. J Exp Med. 1992; 175(5):1381-1390

CD14 --> relevant citations:
1. Spildrejorde M, et al. 2014. Physiol Genomics. 46:512. PubMed
2. Offersen R, et al. 2016. J Virol. 90: 4441 - 4453. PubMed
3. Wang H, et al. 2017. J Neurochem. 141:347. PubMed
4. Miksiunas R, et al. 2020. Int J Mol Sci. 21:00. PubMed
5. Constantinescu-Bercu A, et al. 2020. eLife. 9:00. PubMed

Mouse
CD45 -->relevant citations:
1. Afar B, Merrill J, Clark EA. Detection of lymphocyte subsets using three-color/single-laser flow cytometry and the fluorescent dye peridinin chlorophyll-alpha protein. J Clin Immunol. 1991; 11(5):254-261
2. Greimers R, Trebak M, Moutschen M, Jacobs N, Boniver J. Improved four-color flow cytometry method using fluo-3 and triple immunofluorescence for analysis of intracellular calcium ion ([Ca2+]i) fluxes among mouse lymph node B- and T-lymphocyte subsets. Cytometry. 1996; 23(3):205-217
3. Johnson P, Maiti A, Ng DHW. CD45: A family of leukocyte-specific cell surface glycoproteins. In: Herzenberg LA, Weir DM, Herzenberg LA, Blackwell C , ed. Weir's Handbook of Experimental Immunology, Vol 2. Cambridge: Blackwell Science; 1997; :62.1-62.16
4. Lagasse E, Connors H, Al-Dhalimy M, et al. Purified hematopoietic stem cells can differentiate into hepatocytes in vivo. Nat Med. 2000; 6(11):1212-1213
5. Ledbetter JA, Herzenberg LA. Xenogeneic monoclonal antibodies to mouse lymphoid differentiation antigens. Immunol Rev. 1979; 47:63-90
6. Shapiro HM. Practical Flow Cytometry, 3rd Edition. New York: Wiley-Liss, Inc; 1995; :280-281
7. Thomas ML. The leukocyte common antigen family. Annu Rev Immunol. 1989; 7:339-369
8. Waggoner AS, Ernst LA, Chen CH, Rechtenwald DJ. PE-CY5. A new fluorescent antibody label for three-color flow cytometry with a single laser. Ann N Y Acad Sci. 1993; 677:185-193

CD11b -->relevant citations:
1. Barclay NA, Brown MH, Birkeland ML, et al, ed. The Leukocyte Antigen FactsBook. San Diego, CA: Academic Press; 1997
2. Fleit HB, Wright SD, Unkeless JC. Human neutrophil Fc gamma receptor distribution and structure. Proc Natl Acad Sci U S A. 1982; 79(10):3275-3279
3. Knapp W. W. Knapp .. et al., ed. Leucocyte typing IV : white cell differentiation antigens. Oxford New York: Oxford University Press; 1989; :1-1182
4. Roederer M, Kantor AB, Parks DR, Herzenberg LA. Cy7PE and Cy7APC: bright new probes for immunofluorescence. Cytometry. 1996; 24(3):191-197
5. Stroncek DF, Skubitz KM, Plachta LB, et al. Alloimmune neonatal neutropenia due to an antibody to the neutrophil Fc-gamma receptor III with maternal deficiency of CD16 antigen. Blood. 1991; 77(7):1572-1580
6. van Vugt MJ, van den Herik-Oudijk IE, van de Winkle JG. Binding of PE-CY5 conjugates to the human high-affinity receptor for IgG (CD64). Blood. 1996; 88(6):2358-2361
7. Wirthmueller U, Kurosaki T, Murakami MS, Ravetch JV. Signal transduction by Fc gamma RIII (CD16) is mediated through the

gamma chain. J Exp Med. 1992; 175(5):1381-1390

Ly6G -->relevant citations:
1. Fleming TJ, Fleming ML, Malek TR. Selective expression of Ly-6G on myeloid lineage cells in mouse bone marrow. RB6-8C5 mAb to granulocyte-differentiation antigen (Gr-1) detects members of the Ly-6 family. J Immunol. 1993; 151(5):2399-2408

G-CSF -->validation statements:
This Antibody was verified by Neutralization to ensure that the antibody binds to the antigen stated. Neutralization antibody testing demonstrates the specificity of an antibody through a correlation between antibody binding and the activity of the target.
Neutralization of mouse G-CSF is shown by decrease in CPM (measure of proliferation) with increasing concentrations of G-CSF monoclonal antibody (MA5-23758).

GM-CSF -->relevant citations:
Paschall AV, Zhang R, Qi CF, Bardhan K, Peng L, Lu G, Yang J, Merad M, McGaha T, Zhou G, Mellor A, Abrams SI, Morse HC 3rd, Ozato K, Xiong H, Liu K. IFN regulatory factor 8 represses GM-CSF expression in T cells to affect myeloid cell lineage differentiation. J Immunol. 2015 Mar 1;194(5):2369-79. doi: 10.4049/jimmunol.1402412. Epub 2015 Feb 2. PMID: 25646302; PMCID: PMC4340766.

validation statements:
This Antibody was verified by Neutralization to ensure that the antibody binds to the antigen stated. Neutralization antibody testing demonstrates the specificity of an antibody through a correlation between antibody binding and the activity of the target.
Neutralization of GM-CSF is shown by decrease in RFU (measure of proliferation) with increasing concentrations of GM-CSF monoclonal antibody (MA5-23799).

CXCL-2 --> validation statements:
This Antibody was verified by Neutralization to ensure that the antibody binds to the antigen stated. Neutralization antibody testing demonstrates the specificity of an antibody through a correlation between antibody binding and the activity of the target.
Neutralization of CXCL2/MIP-2 is shown by the decrease in fluorescence (Chemotaxis measured by Resazurin) with increasing concentrations of CXCL2/MIP-2 Antibody (Product # MA5-23737).
relevant citations: Liu, T. et al. Local sympathetic innervations modulate the lung innate immune responses. Sci. Adv. 6, eaay1497 (2020).

CXCL-1 --> validation statements:
This Antibody was verified by Neutralization to ensure that the antibody binds to the antigen stated. Neutralization antibody testing demonstrates the specificity of an antibody through a correlation between antibody binding and the activity of the target.
Neutralization of CXCL1 is shown by the decrease in fluorescence (Chemotaxis measured by Resazurin) with increasing concentrations of CXCL1 Antibody (Product # MA5-23745).
relevant citations: Jackson-Jones, L. H. et al. Stromal Cells Covering Omental Fat-Associated Lymphoid Clusters Trigger Formation of Neutrophil Aggregates to Capture Peritoneal Contaminants. Immunity 52, 700-715.e6 (2020).

CD177 -->relevant citations:
1. Loughner CL, Bruford EA, McAndrews MS, Delp EE, Swamynathan S, Swamynathan SK. Organization, evolution and functions of the human and mouse Ly6/uPAR family genes.. Hum Genomics. 2016; 10:10
2. Xie Q, Klesney-Tait J, Keck K, et al. Characterization of a novel mouse model with genetic deletion of CD177.. Protein Cell. 2015; 6(2):117-26

Ly6G -->relevant citations1.
Davis, R. W. t., et al. (2018). “Luminol Chemiluminescence Reports Photodynamic Therapy-Generated Neutrophil Activity In Vivo and Serves as a Biomarker of Therapeutic Efficacy.” Photochem Photobiol.
2.Moynihan, K. D., et al. (2016). “Eradication of large established tumors in mice by combination immunotherapy that engages innate and adaptive immune responses.” Nat Med. doi: 10.1038/nm.4200
3. Coffelt, S. B., et al. (2015). “IL-17-producing gammadelta T cells and neutrophils conspire to promote breast cancer metastasis.” Nature 522(7556): 345-348

Ly6C --> relevant citations
J1. utila MA, et al. 1988. Eur. J. Immunol. 18:1819. (Activ)
2. Herold KC, et al. 1990. Diabetes 39:815. (Activ)
3. Havran WL, et al. 1988. J. Immunol. 140:1034 (Activ)
4. Flanagan K, et al. 2008. J. Immunol. 180:3874. (IHC)
5. Makaroff LE, et al. 2009. P. Natl. Acad. Sci. USA 106:4799. (FC)

F4/80 --> relevant citations
1. Schaller E, et al. 2002. Mol. Cell. Biol. 22:8035. (IHC)
2. Stevceva L, et al. 2001. BMC Clin Pathol. 1:3. (IHC)
3. Kobayashi M, et al.2008. J. Leukoc. Biol. 83:1354. PubMed
4. Poeckel D, et al. 2009. J. Biol Chem. 284:21077.

CD62L --> relevant citations
1. Cerwenka A, Carter LL, Reome JB, Swain SL, Dutton RW. In vivo persistence of CD8 polarized T cell subsets producing type 1 or type 2 cytokines. J Immunol. 1998; 161(1):97-105. (Biology). View Reference
2. Gallatin WM, Weissman IL, Butcher EC. A cell-surface molecule involved in organ-specific homing of lymphocytes. Nature. 1983; 304(5921):30-34. (Immunogen). View Reference
3. Iwabuchi K, Ohgama J, Ogasawara K, et al. Distribution of MEL-14+ cells in various lymphoid tissues. Immunobiology. 1991; 182(2):161-173. (Biology). View Reference

CD63 --> relevant citations

1. Miyamoto H, Homma M, Hotta H. Molecular cloning of the murine homologue of CD63/ME491 and detection of its strong expression in the kidney and activated macrophages. Biochim Biophys Acta. 1994; 1217(3):312-316. (Biology). View Reference<br>2.Verjan Garcia N, Umemoto E, Saito Y, et al. SIRPalpha/CD172a regulates eosinophil homeostasis. J Immunol. 2011; 187(5):2268-2277. (Immunogen: Flow cytometry, Fluorescence microscopy, Immunofluorescence). View Reference<br><br><br>Rat<br>CD11b -->relevant citations:<br>1. Tamatani T, Kitamura F, Kuida K, et al. Characterization of rat LECAM-1 (L-selectin) by the use of monoclonal antibodies and evidence for the presence of soluble LECAM-1 in rat sera. Eur J Immunol. 1993; 23(9):2181-2188

Eukaryotic cell lines

Policy information about cell lines

Cell line source(s)	CHO cells (ACC-110) were purchased from the Leibnitz Institute DSMZ German Collection of Microorganisms and Cell Cultures.
Authentication	None of the cell lines used were authenticated.
Mycoplasma contamination	All cell lines were negative for mycoplasma contamination.
Commonly misidentified lines<br>(See ICLAC register)	No commonly misidentified cell lines were uses in this study.

Animals and other organisms

Policy information about studies involving animals; ARRIVE guidelines recommended for reporting animal research

Laboratory animals	For animal experiments, male 10 to 12-week-old C57Bl/6 mice ranging from 20 to 30 g body weight (BW) were used. Furthermore, blood samples were anlyzed from male Wistar rats (200–250 g of BW, 12–16 weeks of age) and adult (approx. 1.5 years old) female Aachen minipigs with a mean body weight of 6--70 kg. Animals used in this study were housed at the central animal facility of the Heinrich-Heine-Universität Düsseldorf (ZETT, Düsseldorf, Germany) on a 12 hrs light/dark cycle with 40-60% humidity, fed with a standard chow diet and received tap water ad libitum.
Wild animals	This study did not involve wild animals.
Field-collected samples	The study did not involve samples collected from the field.
Ethics oversight	Animal experiments were performed in accordance with the European Union guidelines described in the directive 2010/63/EU and were approved by North Rhine Westphalian State Agency for Nature, Environment and Consumer Protection (LANUV = Landesamt für Natur, Umwelt und Verbraucherschutz Nordrhein-Westfalen), Germany with the file reference 81-02.04.2017.A468 (mice), 81-02.04. 2020.A290 (mice), L84-02.04.2016.A322 (pig) and 84-02.04.2014.A232 (rat).

Note that full information on the approval of the study protocol must also be provided in the manuscript.

Human research participants

Policy information about studies involving human research participants

Population characteristics	In total blood samples from 17 patients (12/5 male/female) with myocardial infarction (STEMI) were used (age: 66.9±13.6 years). Samples were analyzed 24 hours after myocardial infarction. Tissue samples from the oral cavity were obtained from 6 patients (4/2 female/male, 34.1±21.7 years) and tissue samples from explanted human hearts from 3 patients (n=3, 1/2 female/male, 41.6±18.2 years)
Recruitment	Patients of the University Hospital Düsseldorf were used for this study and all participants gave written informed consent.
Ethics oversight	All studies with human samples were conducted after informed consent according to the declaration of Helsinki and local ethics board approval (Ethikkommission, Universitätsklinikum Düsseldorf, Germany; file references 2017114486 and 2021-1635).

Note that full information on the approval of the study protocol must also be provided in the manuscript.

Flow Cytometry

Plots

Confirm that:

☒ The axis labels state the marker and fluorochrome used (e.g. CD4-FITC).

☒ The axis scales are clearly visible. Include numbers along axes only for bottom left plot of group (a 'group' is an analysis of identical markers).

☒ All plots are contour plots with outliers or pseudocolor plots.

☒ A numerical value for number of cells or percentage (with statistics) is provided.

Methodology

Sample preparation

Mouse blood:
To obtain circulating immune cells, heparinized blood was withdrawn by venous puncture of the inferior vena cava. Blood was collected via a 23G cannula in heparine-aerated collection tubes. Erythrocytes were lysed by adding the 4-fold amount of ammonium chloride buffer (pH 7.4). After 10 min of incubation at room temperature the samples were centrifuged at 350×g for 10 min at 20 °C. For isolation of neutrophils from the bone marrow, mice were sacrificed via cervical dislocation and tibia and femur were dissected. Afterwards, cells were isolated from the bone marrow using a centrifugation dependent protocol. To obtain a pure neutrophil population the EasySepTM mouse neutrophil enrichment kit from StemCell was used.

Mouse matrigel:
Mice were sacrificed by cervical dislocation and the matrigel plug (see below for implantation procedure) was carefully excised. The plug was incubated in DMEM containing 1 mg/ml collagenase II for 10 min at 37 °C. Afterwards the sample was meshed through a cell strainer (40 µm) and isolated cells were resuspended in MACS for further experiments.

Mouse heart:
Mice were sacrificed by cervical dislocation and the thorax was opened. The heart was excised and directly transferred into a 6 cm dish with MACS buffer to flush out the blood. Subsequently, the heart was transferred into DMEM containing 1 mg/ml collagenase and minced into small pieces. Samples were incubated at 37 °C for 30 min under constant shaking and afterwards meshed through a cell strainer (40 µm). To remove cardiomyocytes, samples were centrifuged at 5×g for 5 minutes. The resulting supernatant containing the immune cells was used for further experiments.

Pig and rat blood:
To obtain immune cells from pigs, heparinized blood was withdrawn from the ear vein of the animals using a 22G vein catheter as previously described5. For rats, heparinized blood was withdrawn by cardiac puncture of the left ventricle via a 23G cannula in heparine-aerated collection tubes. For both species, erythrocytes were lysed by adding the 4-fold amount of ammonium chloride buffer (pH 7.4) to the blood. After 10 min of incubation at room temperature the samples were centrifuged at 350×g for 10 min at 20 °C.

Instrument

FACS Canto II, LSR Fortessa

Software

FACS Diva, FlowJo

Cell population abundance

No cell sorting was used within this study.

Gating strategy

Cells were gated with appropriate forward/side scatter settings and thresholds for excluding debris. To omit dead cells, samples were stained with 1 µg/ml DAPI (4′,6-Diamidin-2-phenylindol, Merck). For analysis, cells were gated with FACS Diva software and the mean fluorescence intensities and/or the number of positive cells were determined, depending on the experiment.

Human samples:
The individual human immune cell populations were discriminated by antibody staining against CD45 (Biolegend, clone HI30), CD11b (BD Biosciences, clone ICRF44) and CD16 (BD Biosciences, clone 3G8); lymphocytes: CD45+, CD11b-, CD16-; monocytes: CD45+, CD11b+, CD16-; neutrophil granulocytes: CD45+, CD11b+, CD16+.

Murine samples:
The individual mouse immune cell populations were discriminated by antibody staining against CD45 (BD Biosciences, clone 30-F11), CD11b (Biolegend, clone M1/70) and Ly6G (BD Biosciences, clone 1A8); lymphocytes: CD45+, CD11b-, Ly6G-; monocytes: CD45+, CD11b+, Ly6G-; neutrophil granulocytes: CD45+, CD11b+, Ly6G+. Cells were stained for 20 min at 4 °C.

☒ Tick this box to confirm that a figure exemplifying the gating strategy is provided in the Supplementary Information.
